# Interface atom mobility and charge transfer effects on CuO and Cu_2_O formation on Cu_3_Pd(111) and Cu_3_Pt(111)

**DOI:** 10.1038/s41598-021-82180-w

**Published:** 2021-02-15

**Authors:** Yasutaka Tsuda, Jessiel Siaron Gueriba, Takamasa Makino, Wilson Agerico Diño, Akitaka Yoshigoe, Michio Okada

**Affiliations:** 1 Department of Chemistry, Osaka University, Toyonaka, Osaka 560-0043 Japan; 2 Materials Sciences Research Center, Japan Atomic Energy Agency, 1-1-1 Kouto, Sayo-cho, Sayo-gun, Hyogo, 679-5148 Japan; 3 Department of Applied Physics, Osaka University, Suita, Osaka 565-0871 Japan; 4 Department of Physics, De La Salle University, 2401 Taft Avenue, Manila, 0922 Philippines; 5 Center for Atomic and Molecular Technologies, Osaka University, Suita, Osaka 565-0871 Japan; 6 Institute for Radiation Sciences, Osaka University, Toyonaka, Osaka 560-0043 Japan

**Keywords:** Physical chemistry, Surface chemistry

## Abstract

We bombarded $$\mbox{$\text{Cu}_{3}\text{Pd}(111)$}$$ and $$\mbox{$\text{Cu}_{3}\text{Pt}(111)$}$$ with a 2.3 eV hyperthermal oxygen molecular beam (HOMB) source, and characterized the corresponding (oxide) surfaces with synchrotron-radiation X-ray photoemission spectroscopy (SR-XPS). At $$300\,\text{K}$$, CuO forms on both $$\mbox{$\text{Cu}_{3}\text{Pd}(111)$}$$ and $$\mbox{$\text{Cu}_{3}\text{Pt}(111)$}$$. When we increase the surface temperature to $$500\,\text{K}$$, $$\mbox{$\text{Cu}_{2}\text{O}$}$$ also forms on $$\mbox{$\text{Cu}_{3}\text{Pd}(111)$}$$, but not on $$\mbox{$\text{Cu}_{3}\text{Pt}(111)$}$$. For comparison, $$\mbox{$\text{Cu}_{2}\text{O}$}$$ forms even at $$300\,\text{K}$$ on Cu(111). On $$\mbox{$\text{Cu}_{3}\text{Au}(111)$}$$, $$\mbox{$\text{Cu}_{2}\text{O}$}$$ forms only after $$500\,\text{K}$$, and no oxides can be found at $$300\,\text{K}$$. We ascribe this difference in Cu oxide formation to the mobility of the interfacial species (Cu/Pd/Pt) and charge transfer between the surface Cu oxides and subsurface species (Cu/Pd/Pt).

## Introduction

Metal oxides have long attracted researchers’ attention, particularly the role of metal valence electrons in determining structure and properties^1,2^. Manganese (Mn) in Mn oxides, for example, can take more than one oxidation state (mixed valency). $${\text {MnO}}_{x}$$ exhibit better catalytic activity than MnO^3^. Copper (Cu) oxides also attract particular attention, due to their utility, industrial applications, abundance, low-cost, and non-toxicity. $$\mbox{$\text{Cu}_{2}\text{O}$}$$ and CuO, two of the most common forms of Cu oxides, find applications as anodes in lithium ion battery^4^ and solar cells^5^. Copper oxides are also expected to be used as catalysts for CO^6^, NO dissociation^7^, adsorption of $${\text {H}}_{2}$$^8^, $${\text {O}}_{2}$$^9–11^, and $$\mbox{$\text{H}_{2}\text{O}$}$$^12,13^. Similarly, the catalytic reactivities of Cu oxides vary with the Cu oxidation state. Several factors determine oxide formation on the surface, e.g., translational and internal (vibration and rotation) energies of impinging $${\text {O}}_{2}$$, surface temperature, and surface electronic state^14^. Alloying allows for a simple way to vary the surface electronic state and the corresponding reactivity. For example, by alloying Cu with gold (Au) and tuning the surface composition, previous studies tried to enhance the activity and selectivity of Cu–Au nanoparticle catalysts for $${\text {CO}}_{2}$$ reduction^15,16^. On $$\mbox{$\text{Cu}_{3}\text{Au}(111)$}$$, the presence of (the “inert”) Au hinders $${\text {O}}_{2}$$ dissociative adsorption, as compared to Cu(111)^17–20^. At surface temperature $$T_{{\rm S}} = 300\,\text{K}$$, Au-rich layers, formed between the bulk and surface, prevents Cu oxide formation further into bulk. Atom diffusion at $$T_{\rm S} = 500\,\text{K}$$ promotes $$\mbox{$\text{Cu}_{2}\text{O}$}$$ formation, but leaves a protective Au-rich layer that prevents further oxidation into the bulk. On the other hand, Cu–palladium (Pd) alloys synergetically enhanced $${\text {CO}}_{2}$$ hydrogenation to alcohol, as compared to mono-metallic catalysts^21,22^. Cu–platinum (Pt) alloys, viz., $$\mbox{$\text{Pt}_{34.5}\text{Cu}_{65.5}$}$$, also showed superior methanol oxidation and oxygen reduction performance^23^. Moreover, single Pt atoms at the Cu metal-oxide interface promote the reduction of $$\mbox{$\text{Cu}_{2}\text{O}$}$$ by $${\text {H}}_{2}$$ exposure^24^. These results indicate that choosing the right alloy components strongly affect reactivity. Here, we report on how alloying Cu with Pd and Pt, elements known to be more reactive than Au, affects oxidation of the corresponding surfaces. We bombarded $$\mbox{$\text{Cu}_{3}\text{Pd}(111)$}$$ and $$\mbox{$\text{Cu}_{3}\text{Pt}(111)$}$$ with a 2.3 eV hyperthermal oxygen molecular beam (HOMB) source, and characterized the corresponding (oxidized) surfaces using X-ray photoemission spectroscopy (XPS) in conjunction with synchrotron radiation (SR). We found that the presence of interfacial Pd and Pt, between surface Cu oxides and bulk, suppresses atomic diffusion and induces charge transfer near the interface. These result in the formation of protective layers that prevent Cu oxidation further into the bulk. These also account for the difference in Cu valencies and resulting oxidation states (Cu oxide species formed) on $$\mbox{$\text{Cu}_{3}\text{Pd}(111)$}$$ and $$\mbox{$\text{Cu}_{3}\text{Pt}(111)$}$$, as compared to Cu(111) and $$\mbox{$\text{Cu}_{3}\text{Au}(111)$}$$.

## Results

### Pd and Pt (surface) concentration profiles

In Table 1, we show the corresponding experimental and calculated layer concentration profiles $$x_{n}$$ (in units of $$\%$$-Pd or $$\%$$-Pt) for clean $$\mbox{$\text{Cu}_{3}\text{Pd}(111)$}$$ and $$\mbox{$\text{Cu}_{3}\text{Pt}(111)$}$$. To experimentally determine the corresponding Pd and Pt layer concentrations $$x_{n}$$, of the *n*th layer from the vacuum, we followed the method described in previous studies^17^, using the photoelectron detection angle dependence of the intensities of the bulk (B-) and surface (S-) components of the Pd-3*d* and Pt-4*f* XPS spectra. (For more details on the procedures, analyses, and calculations, cf., Section S.1 in the supplementary information.)

**Figure 1:**
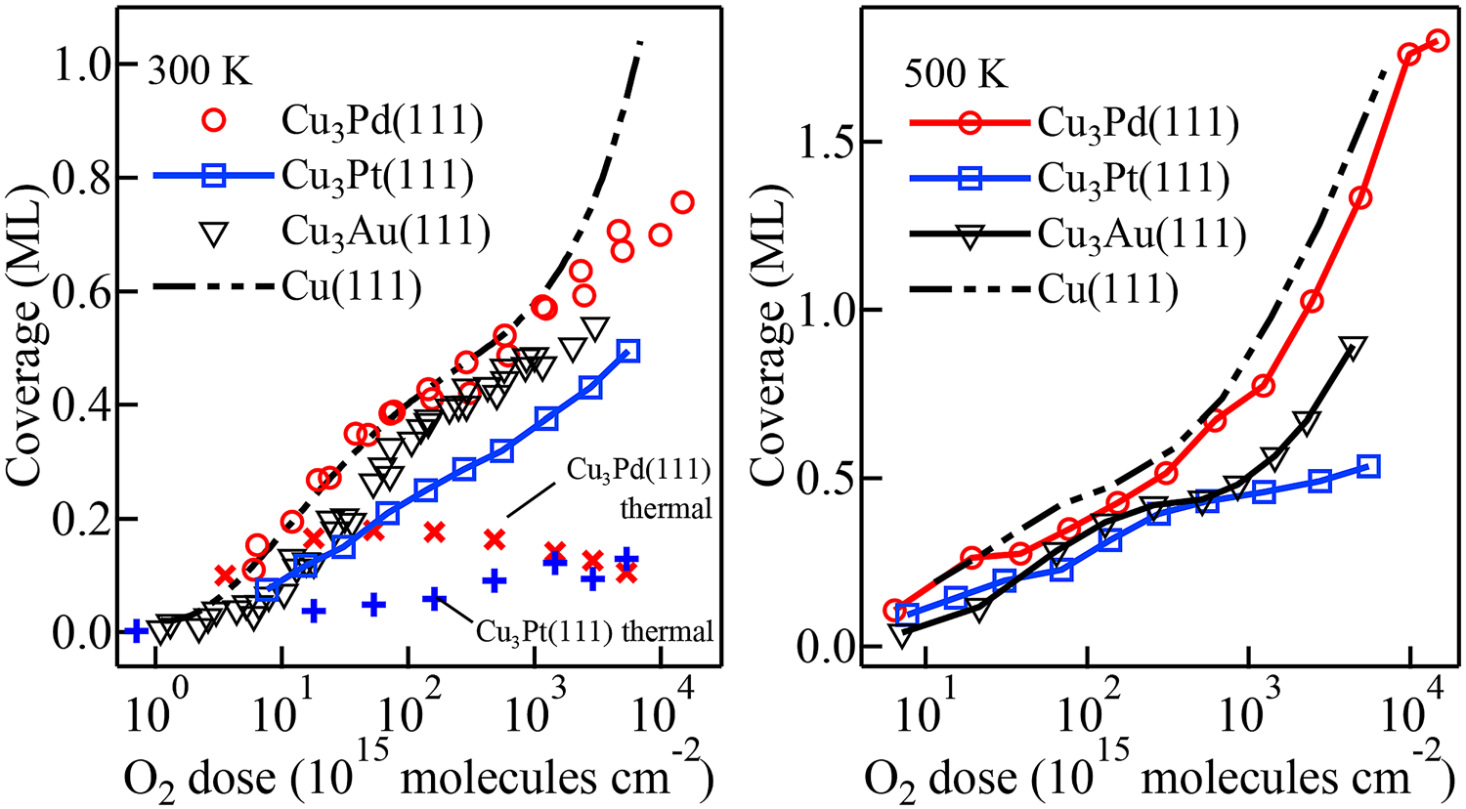
O uptake curves for $$\mbox{$\text{Cu}_{3}\text{Pd}(111)$}$$ and $$\mbox{$\text{Cu}_{3}\text{Pt}(111)$}$$ after 2.3 eV HOMB irradiation at $$T_{\rm S} = 300$$ and $$500\,\text{K}$$. For reference, O uptake curves taken after thermal $${\text {O}}_{2}$$ exposure, and O uptake curves for Cu(111) and $$\mbox{$\text{Cu}_{3}\text{Au}(111)$}$$ after 2.3 eV HOMB irradiation, are also shown.

On the $$\mbox{$\text{Cu}_{3}\text{Pd}(111)$}$$, our experimental analyses (calculation results) show a Pd concentration of $$x_{1}$$= 44 (50) (cf., first two rows, Table 1), which is greater than the bulk value of $$x_{\rm bulk} = 25$$. This indicates Pd segregation onto the surface, resulting in a Pd-rich surface.

On the $$\mbox{$\text{Cu}_{3}\text{Pt}(111)$}$$, our experimental analyses show $$x_{1}$$ = 23, $$x_{2}$$ = 33, and $$x_{3}$$ = 88 (cf., third row, Table 1). This indicates Pt segregation onto the 2nd and 3rd layers (Pt-rich 2nd and 3rd layers). Previous studies using low energy ion scattering (LEIS) ^25^ report similar results, viz., $$x_{1}$$ = 20, $$x_{2}$$ = 31 (cf., fourth row, Table 1). For comparison, low energy electron diffraction (LEED) studies^26^ report $$x_{1}$$ = 28, $$x_{2}$$ = 8, $$x_{3}$$ = 48, and $$x_{4}$$ = 8 (cf., fifth row, Table 1). On the other hand, calculation results show $$100\%$$-Pt segregation onto the 1st (surface) layer, and $$50\%$$ Pt segregation onto the 3rd layer. Regardless, both experiment and theory show (on the average) similar Pt concentrations from the 1st to the 3rd layer, viz., $$48\%$$ and $$50\%$$, respectively.

**Table 1:** Layer concentration profiles $$x_{n}$$ (in units of $$\%$$-Pd or $$\%$$-Pt) for clean $$\mbox{$\text{Cu}_{3}\text{Pd}(111)$}$$ and $$\mbox{$\text{Cu}_{3}\text{Pt}(111)$}$$.

Sample		1st layer	2nd layer	3rd layer	4th layer
		$x_{1}$	$x_{2}$	$x_{3}$	$x_{4}$
$$\mbox{$\text{Cu}_{3}\text{Pd}(111)$}$$	Experiment$${}^{{\rm a}}$$	44	22	Bulk(25)	Bulk(25)
	Theory^a^	50	25	Bulk(25)	Bulk(25)
$$\mbox{$\text{Cu}_{3}\text{Pt}(111)$}$$	Experiment^a^	23	33	88	20
	Experiment^25^	20	31	Bulk(25)	Bulk(25)
	Experiment^26^	28	8	48	8
	Theory^a^	100	0	50	Bulk(25)

^a^This work.

**Table 2:** Oxides species formed on Cu and Cu-based alloys after 2.3 eV HOMB irradiation at $T_{{\rm S}}=300\,\text{K}$.

	$$\mbox{$\text{Cu}_{3}\text{Pd}(111)$}$$	$$\mbox{$\text{Cu}_{3}\text{Pt}(111)$}$$	Cu(111)^14, 15^	$$\mbox{$\text{Cu}_{3}\text{Au}(111)$}$$^17–20^
300 K	CuO	CuO	${\text {Cu}}_{2}$O	Only chemisorbed O
500 K	${\text {Cu}}_{2}$O	CuO	${\text {Cu}}_{2}$O	${\text {Cu}}_{2}$O

### LEED patterns

The clean $$\mbox{$\text{Cu}_{3}\text{Pd}(111)$}$$ exhibits a ($$2 \times 2$$) LEED pattern with some spot splittings (cf., Fig. S.4(a) in the supplementary information), which we ascribe to the presence of structural anti-phase domains on the surface (considering the $$44\%$$-Pd surface segregation and three rotationally symmetric domains). For comparison, the clean $$\mbox{$\text{Cu}_{3}\text{Pt}(111)$}$$ exhibits a ($$1 \times 1$$) LEED pattern, indicating random distribution of $$23\%$$-Pt atoms on the 1st layer.

### Oxygen uptake curves

In Fig. 1, we show the O uptake curves for $$\mbox{$\text{Cu}_{3}\text{Pd}(111)$}$$ and $$\mbox{$\text{Cu}_{3}\text{Pt}(111)$}$$ produced by integrating a series of O-1*s* XPS spectra measured after 2.3 eV HOMB irradiation at $$T_{\rm S} = 300$$ and $$500\,\text{K}$$ (also cf., Fig. S.6 in supplementary information). We determined the oxygen coverage by comparing Cu(100)-$$(2\sqrt{2} \times \sqrt{2})$$-O and Cu(110)-(2 $$\times$$ 1)-O^27^ and also cross-checked by using the intensity ratios of O-1*s*/Pd-3*d* and O-1*s*/Pt-4*f*. For comparison, we also show the O uptake curves taken after thermal $${\text {O}}_{2}$$ exposure, and the O uptake curves for Cu(111) and $$\mbox{$\text{Cu}_{3}\text{Au}(111)$}$$ taken after 2.3 eV HOMB irradiation. Comparing the results of HOMB irradiation and thermal $${\text {O}}_{2}$$ exposure, we confirm the need for higher translational energies to realize effective oxide formation. However, following procedures previously reported for determining Au concentrations on the oxidized $$\mbox{$\text{Cu}_{3}\text{Au}(111)$}$$^17^, we were not able to consistently obtain the Pd and Pt concentrations. This may be due to the presence of Cu oxide islands on the corresponding surfaces, whereas only homogeneous adsorbed-O forms on $$\mbox{$\text{Cu}_{3}\text{Au}(111)$}$$^17,28^.

### Cu-$L_{3}M_{4,5}M_{4,5}$ and Cu-2*p* spectra

**Figure 2:**
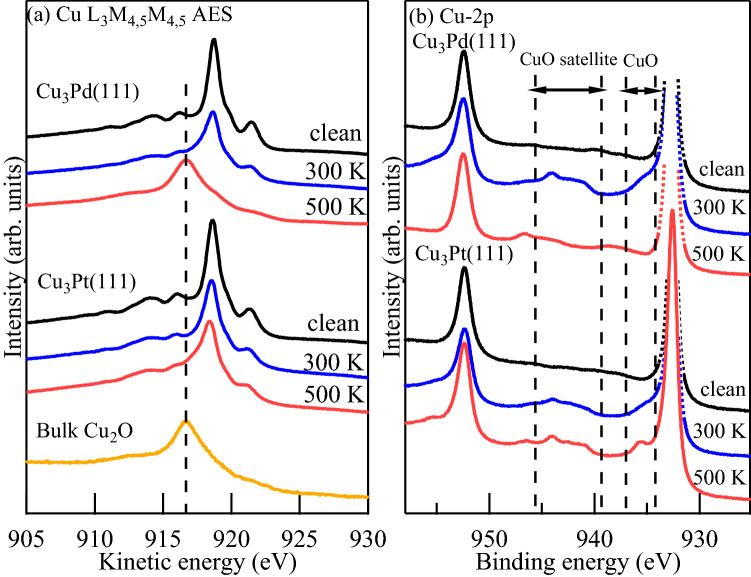
(**a**) Cu-$$L_{3}M_{4,5}M_{4,5}$$ AES and (**b**) Cu-2*p* XPS spectra of $$\mbox{$\text{Cu}_{3}\text{Pd}(111)$}$$ and $$\mbox{$\text{Cu}_{3}\text{Pt}(111)$}$$, taken after 2.3 eV HOMB irradiation at $$T_{\rm S}= 300\,\text{K}$$ and $$500\,\text{K}$$, at a photoelectron detection angle $$\theta =70^{\circ }$$ from the surface normal. For $$\mbox{$\text{Cu}_{3}\text{Pd}(111)$}$$, corresponding AES spectra for O-coverages of 0 ML (clean), 0.76 ML at $$300\,\text{K}$$, and 1.8 ML at $$500\,\text{K}$$, are shown. Similarly, for $$\mbox{$\text{Cu}_{3}\text{Pt}(111)$}$$, corresponding AES spectra for O-coverages of 0 ML (clean), 0.49 ML at $$300\,\text{K}$$, 0.53 ML at $$500\,\text{K}$$, are shown. Bulk $$\mbox{$\text{Cu}_{2}\text{O}$}$$ spectra also shown in (**a**) for reference, with dashed vertical line indicating characteristic $$\mbox{$\text{Cu}_{2}\text{O}$}$$ peak position.

In Fig. 2, we show the Cu-$$L_{3}M_{4,5}M_{4,5}$$ AES (Auger electron spectroscopy) and Cu-2*p* XPS spectra of the corresponding Cu oxides formed on $$\mbox{$\text{Cu}_{3}\text{Pd}(111)$}$$ and $$\mbox{$\text{Cu}_{3}\text{Pt}(111)$}$$. For reference, note the $$\mbox{$\text{Cu}_{2}\text{O}$}$$ features appearing at ca. 917 eV kinetic energy (cf., bulk $$\mbox{$\text{Cu}_{2}\text{O}$}$$ peak, Fig. 2a). From Fig. 2a, we find prominent $$\mbox{$\text{Cu}_{2}\text{O}$}$$ formation only for $$\mbox{$\text{Cu}_{3}\text{Pd}(111)$}$$ oxidized at $$T_{\rm S} = 500\,\text{K}$$. On the other hand, in Fig. 2b, we find characteristics of CuO (cf., shoulder at ca. 936 eV and satellite peaks between ca. 939 eV and 946 eV^29^) for both $$\mbox{$\text{Cu}_{3}\text{Pd}(111)$}$$ and $$\mbox{$\text{Cu}_{3}\text{Pt}(111)$}$$ oxidized at $$T_{\rm S} = 300\,\text{K}$$. We also see that these CuO features persist at $$T_{\rm S} = 500\,\text{K}$$ for $$\mbox{$\text{Cu}_{3}\text{Pt}(111)$}$$, but disappear for $$\mbox{$\text{Cu}_{3}\text{Pd}(111)$}$$. On $$\mbox{$\text{Cu}_{3}\text{Pt}(111)$}$$, AES spectra analyses indicate CuO formation only, both at $$T_{\rm S} = 300\,\text{K}$$ and $$500\,\text{K}$$. On $$\mbox{$\text{Cu}_{3}\text{Pd}(111)$}$$, AES spectra analyses indicate CuO formation at $$T_{\rm S} = 300\,\text{K}$$ and $$\mbox{$\text{Cu}_{2}\text{O}$}$$ formation at $$T_{\rm S} = 500\,\text{K}$$. (Pd-3*d* and Pt-4*f* XPS spectra analyses also indicate that only Cu oxidation occurs on both $$\mbox{$\text{Cu}_{3}\text{Pd}(111)$}$$ and $$\mbox{$\text{Cu}_{3}\text{Pt}(111)$}$$, cf., Section S.4 in supplementary information). In Table 2, we show a summary of oxides formed on the Cu and Cu-based alloy surfaces studied.

**Figure 3:**
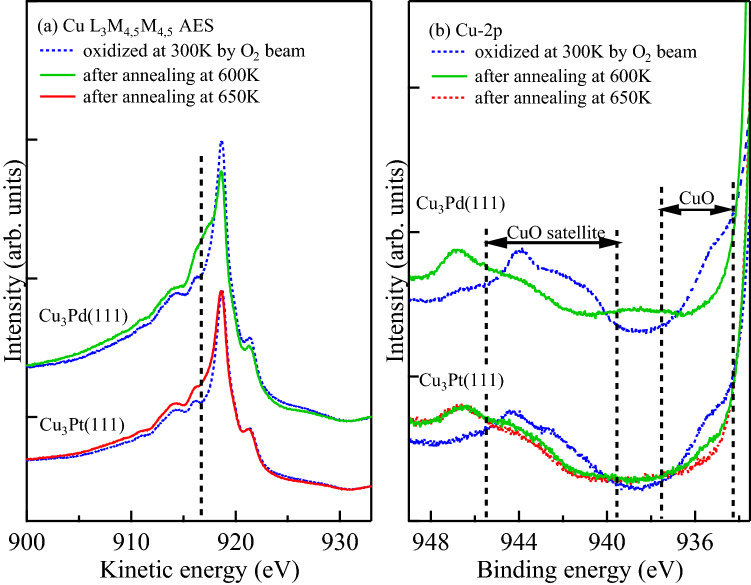
(**a**) Cu-$$L_{3}M_{4,5}M_{4,5}$$ AES and (**b**) Cu-2*p* spectra of $$\mbox{$\text{Cu}_{3}\text{Pd}(111)$}$$ and $$\mbox{$\text{Cu}_{3}\text{Pt}(111)$}$$, taken after 2.3 eV HOMB irradiation at $$T_{\rm S} = 300\,\text {K}$$, and after annealing at $$600\,\text{K}$$ and $$650\,\text{K}$$, at a photoelectron detection angle of $$70^{\circ }$$ from the surface normal. $$\mbox{$\text{Cu}_{3}\text{Pd}(111)$}$$ has 0.50 ML CuO-coverage and $$\mbox{$\text{Cu}_{3}\text{Pt}(111)$}$$ has ca. 0.50 ML CuO-coverage, before annealing. Characteristic $$\mbox{$\text{Cu}_{2}\text{O}$}$$ peak position indicated as dashed vertical line in (**a**), for reference.

In Fig. 3, we show the effect of annealing on the corresponding Cu-$$L_{3}M_{4,5}M_{4,5}$$ AES and Cu-2*p* spectra of $$\mbox{$\text{Cu}_{3}\text{Pd}(111)$}$$ and $$\mbox{$\text{Cu}_{3}\text{Pt}(111)$}$$ oxidized at $$T_{\rm S} = 300\,\text{K}$$. We see enhanced $$\mbox{$\text{Cu}_{2}\text{O}$}$$ features from the Cu-$$L_{3}M_{4,5}M_{4,5}$$ spectra, and diminished CuO features from the Cu-2*p*. (The fitted O-1*s* XPS spectra also show enhanced $$\mbox{$\text{Cu}_{2}\text{O}$}$$ features and diminished CuO features, cf., Fig. S.9 in supplementary information). This suggests the reduction of CuO into $$\mbox{$\text{Cu}_{2}\text{O}$}$$ at high $$T_{\rm S}$$. However, a lingering CuO peak remains on $$\mbox{$\text{Cu}_{3}\text{Pt}(111)$}$$, even after the annealing at $$T_{\rm S} = 600\,\text{K}$$, which we no longer see on $$\mbox{$\text{Cu}_{3}\text{Pd}(111)$}$$. We found that annealing at $$T_{\rm S} = 650\,\text{K}$$ completes the reduction from CuO to $$\mbox{$\text{Cu}_{2}\text{O}$}$$ on the $$\mbox{$\text{Cu}_{3}\text{Pt}(111)$}$$. Therefore, we conclude more stable CuO formation on $$\mbox{$\text{Cu}_{3}\text{Pt}(111)$}$$ as compared to $$\mbox{$\text{Cu}_{3}\text{Pd}(111)$}$$. Similar effect can be observed for Cu(410) oxidation at lower $$T_{\rm S}$$. After 2.2 eV HOMB irradiation, CuO forms on Cu(410) at $$T_{\rm S} = 100\,\text{K}$$^30^. Annealing at $$T_{\rm S} = 273\,\text{K}$$ reduces CuO into $$\mbox{$\text{Cu}_{2}\text{O}$}$$. Alloying increases the corresponding transition temperature from CuO to $$\mbox{$\text{Cu}_{2}\text{O}$}$$. CuO persists even at $$T_{\rm S} = 300\,\text{K}$$.

## Discussions

### $${\text {O}}_{2}$$ Dissociation, CuO and $$\mbox{$\text{Cu}_{2}\text{O}$}$$ formation on Cu and Cu alloys in the early stage of oxidation

The position of the surface *d*-band center relative to the Fermi level^31,32^ has often been used to qualitatively discuss reactivity of dissociative adsorption. The deeper the surface *d*-band center, the less reactive the surface. From XPS measurements, in order of increasing depth with respect to the vacuum level (viz., in order of decreasing reactivity), we have $$\mbox{$\text{Cu}_{3}\text{Pd}$}$$ ($$-2.7~\text {eV}$$), $$\mbox{$\text{Cu}_{3}\text{Pt}$}$$ ($$-2.9~\text {eV}$$), Cu ($$-3.1~\text {eV}$$), and finally $$\mbox{$\text{Cu}_{3}\text{Au}$}$$ ($$-3.7~\text {eV}$$) (also cf., Fig. S.10 in supplementary information, and Ref.^33^). However, the uptake curves in Fig. 1 show different tendency of reactivity. We see O-coverages lower than what we would expect from the *d*-band for both $$\mbox{$\text{Cu}_{3}\text{Pd}(111)$}$$ and $$\mbox{$\text{Cu}_{3}\text{Pt}(111)$}$$.

**Figure 4:**
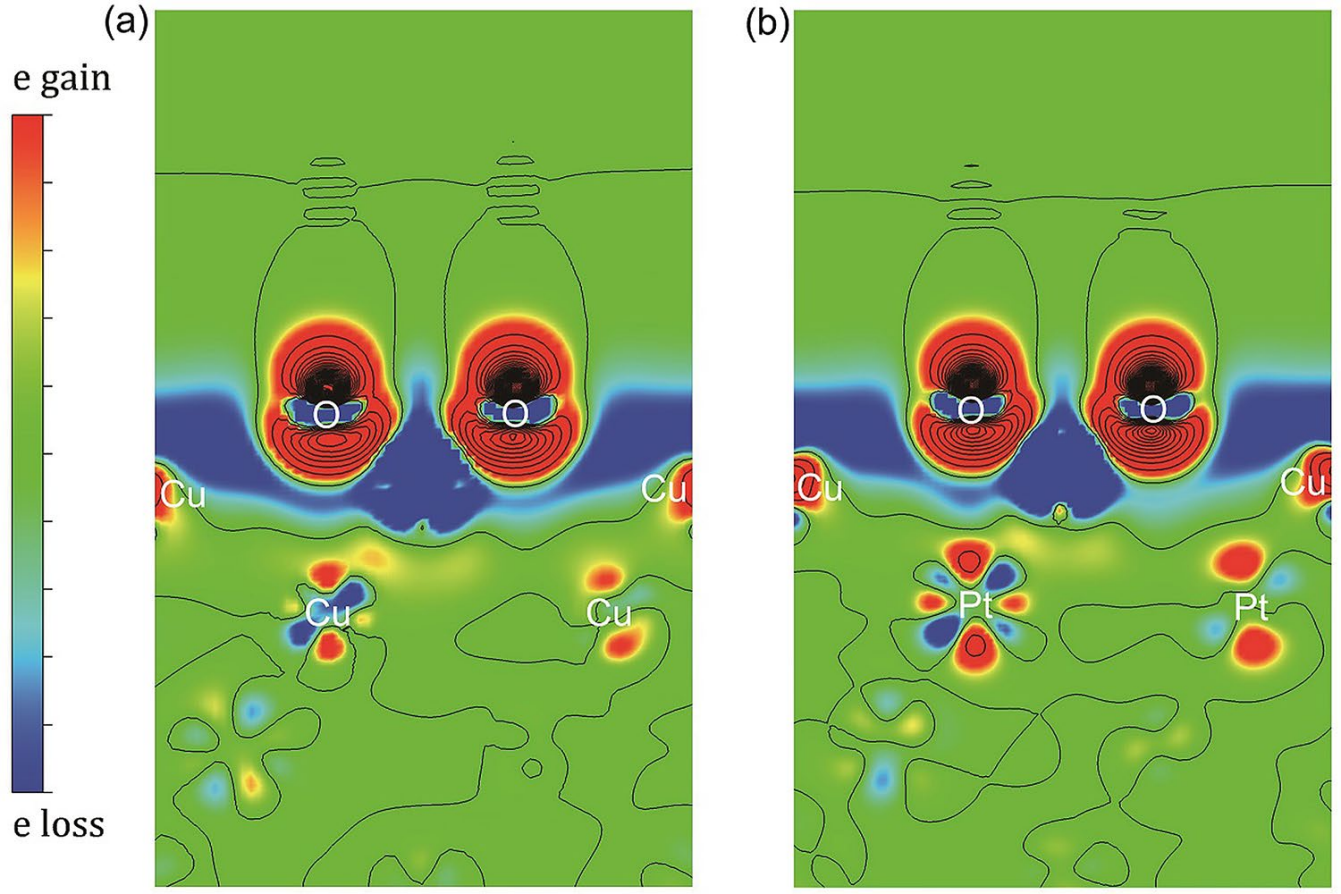
Calculated charge distribution difference for 0.5 ML-O adsorbed on $$\mbox{$\text{Cu}_{3}\text{Pt}(111)$}$$, for layer concentrations (**a**) ($$x_1, x_2, x_{3-7}$$) = (0, 0, 25) and (**b**) ($$x_1, x_2, x_{3-7}$$) = (0, 100, 25). We obtained (**a**) and (**b**) by subtracting the charge distributions of bulk slab and O atoms from that of O-adsorbed slab. We can see a relatively higher electron gain around Pt in the second layer (**b**) than that around Cu (**a**). The electronegative O atoms are relatively far from the Pt subsurface layer. Thus, we associate the charge gain of Pt with the charge loss of surface Cu atoms.

Now, let us consider what the uptake curves taken at $$T_{\rm S} = 300\,\text{K}$$ and $$500\,\text{K}$$ tell us (cf., Fig. 1). Here, we discuss the early stage of oxidation where only the dissociative adsorption of $${\text {O}}_2$$ occurs on Cu(111). Note that on Cu(111) and $$\mbox{$\text{Cu}_{3}\text{Au}(111)$}$$, previous HOMB irradiation studies at 2.3 eV^34^ report that direct $${\text {O}}_{2}$$ dissociative adsorption dominates at the early stage of oxidation, and Cu oxide ($$\mbox{$\text{Cu}_{2}\text{O}$}$$) subsequently forms above $$\sim 10^{17} \text {molecules} \, {\text {cm}}^{-2}$$. In comparison, $$\mbox{$\text{Cu}_{3}\text{Pd}(111)$}$$ and $$\mbox{$\text{Cu}_{3}\text{Pt}(111)$}$$ show Cu oxide (CuO) formation above ca. 8–$$60 \times 10^{15}~\text {molecules}\,{\text {cm}}^{-2}$$ as shown in Fig. S.8 in supplementary information. In other words, Cu oxide forms earlier on $$\mbox{$\text{Cu}_{3}\text{Pd}(111)$}$$ and $$\mbox{$\text{Cu}_{3}\text{Pt}(111)$}$$ than on Cu(111). Taking this into account, we can interpret the reactivity below $$\sim 10^{17} \, \text {molecules}\,{\text {cm}}^{-2}$$ as follows. The inactive CuO formed at an early stage of oxidation makes $$\mbox{$\text{Cu}_{3}\text{Pd}(111)$}$$ and $$\mbox{$\text{Cu}_{3}\text{Pt}(111)$}$$ more robust to further oxidation of bulk Cu, while further bulk Cu is lost through $$\mbox{$\text{Cu}_{2}\text{O}$}$$ formation on Cu(111). The earlier CuO formation at ca. $$8\times 10^{15}~\text {molecules}\,{\text {cm}}^{-2}$$ further indicates a more robust $$\mbox{$\text{Cu}_{3}\text{Pt}(111)$}$$ than $$\mbox{$\text{Cu}_{3}\text{Pd}(111)$}$$. So, based on the corresponding O uptake curves, we have Cu(111), $$\mbox{$\text{Cu}_{3}\text{Pd}(111)$}$$, $$\mbox{$\text{Cu}_{3}\text{Au}(111)$}$$^20^, and $$\mbox{$\text{Cu}_{3}\text{Pt}(111)$}$$, in order of decreasing susceptibility to Cu loss (i.e., $$\mbox{$\text{Cu}_{2}\text{O}$}$$ formation).

### The protective layer

The difference in efficiency of oxide formation between Cu and Cu alloy surfaces can be ascribed to the resulting protective layer of Pd (Pt) layer formed at the interface between the bulk and surface Cu oxide (cf., e.g., $$\mbox{$\text{Cu}_{3}\text{Au}(111)$}$$^20^). As mentioned in S.4 in supplementary information, only Cu oxidation occurs on $$\mbox{$\text{Cu}_{3}\text{Pd}(111)$}$$ and $$\mbox{$\text{Cu}_{3}\text{Pt}(111)$}$$. Previous studies also show that only Cu oxide forms on Cu-Pt alloy^28^ and Cu deposited on Pt(111)^35^. The selective Cu oxidation results in Pd- and Pt-rich interface layers. We see a steep O uptake curve, coming from $$\mbox{$\text{Cu}_{2}\text{O}$}$$ formation^34^, for Cu(111) at $$T_{\rm S} = 300\,\text{K}$$ (cf., Fig. 1 (left-panel), region above ca. $$10^{17}~\text {molecules}\,{\text {cm}}^{-2}$$). On Cu(111), $$\mbox{$\text{Cu}_{2}\text{O}$}$$ formation occurs due to collision induced absorption (CIA)^36^. On $$\mbox{$\text{Cu}_{3}\text{Au}$}$$, the inert Au interface layer prevents O atoms from diffusing further into the bulk by CIA process. As a result, Cu oxide hardly forms at $$300\,\text{K}$$^17–20^. Here, we find that the interface Pd- and Pt- layers also prevent O diffusion further into the bulk to realize the CIA process. It also prevents Cu diffusion from the bulk to the surface CuO.

### Mobility/diffusion

At $$T_{\rm S} = 500\,\text{K}$$, we expect that the increased temperature would enhance atom diffusion, allowing for Cu oxide formation further into the bulk. On $$\mbox{$\text{Cu}_{3}\text{Au}(111)$}$$, $$\mbox{$\text{Cu}_{2}\text{O}$}$$ forms at $$500\,\text{K}$$^19^. $$\mbox{$\text{Cu}_{3}\text{Pd}(111)$}$$ also has a steeper O uptake curve at $$500\,\text{K}$$ than at $$300\,\text{K}$$ (cf., above $$10^{17}~\text {molecules}\,{\text {cm}}^{-2}$$ in Fig. 1). However, $$\mbox{$\text{Cu}_{3}\text{Pt}(111)$}$$ remains relatively inactive, even at $$500\,\text{K}$$. This is because of the presence of less mobile Pt at the interface. In Cu, Pd has an activation/diffusion barriers of ca. 0.88 eV^37^, Au has 1.1 eV^38^, and Pt has 1.51 eV^39^. This is consistent with the reactivity observed above ca. $$10^{17}~\text {molecules}\,{\text {cm}}^{-2}$$ at $$500\,\text{K}$$, i.e., the region where mainly CuO forms. The interface Pt suppresses Cu oxide growth into the bulk even at $$723\,\text{K}$$^28^. Cu diffusion through the Pd (Pt) interface would also be unlikely considering the high Cu diffusion barriers (ca. 2.5 eV in Pd, 2.75 eV in Pt, and 2.0 eV in Au)^40^. As expected, these differences in diffusion barriers affect the kind of Cu oxides formed on the Cu alloy surfaces.

At $100\,\text{K}$, metastable CuO forms on Cu(410)^30^. The low temperature suppresses O diffusion from the surface to the bulk (and also Cu diffusion from the bulk to the surface), while collision induced absorption (CIA) allows for a continuous supply of O atoms to the surface. We can expect similar effects on the Cu-Pd and Cu-Pt alloy surfaces.

At $$300\,\text{K}$$, the presence of Pd or Pt at the corresponding interfaces suppresses the diffusion of O and Cu, while CIA allows for a continuous supply of O atoms on the surface. Similarly, on $$\mbox{$\text{Cu}_{3}\text{Au}(111)$}$$ at 300 K, O atoms adsorbed on surface cannot diffuse into bulk due to the interface Au^17,18^. As a result, no Cu oxides form on $$\mbox{$\text{Cu}_{3}\text{Au}(111)$}$$ at $$300\,\text{K}$$.

At $$500\,\text{K}$$, enhanced diffusion allows oxidation further into the bulk of $$\mbox{$\text{Cu}_{3}\text{Au}(111)$}$$ and $$\mbox{$\text{Cu}_{3}\text{Pd}(111)$}$$, and we find growth of the thermodynamically more stable $$\mbox{$\text{Cu}_{2}\text{O}$}$$. On the other hand, CuO persists on $$\mbox{$\text{Cu}_{3}\text{Pt}$}$$ at $$500\,\text{K}$$ because of the higher Pt diffusion barrier. As shown in Fig. 3, $$\mbox{$\text{Cu}_{2}\text{O}$}$$ forms after annealing (after the HOMB irradiation at $$300\,\text{K}$$). Higher $$T_{\rm S}$$ enables O diffusion further into the bulk and further Cu supply to the surface CuO. This occurs at $$T_{\rm S} = 650\,\text{K}$$ on $$\mbox{$\text{Cu}_{3}\text{Pt}$}$$, and $$T_{\rm S} = 600\,\text{K}$$ on $$\mbox{$\text{Cu}_{3}\text{Pd}$}$$. The less diffusive Pt present at the interface prevents further $$\mbox{$\text{Cu}_{2}\text{O}$}$$ formation as compared to Pd. At $$T_{\rm S} = 723\,\text{K}$$, $$\mbox{$\text{Cu}_{2}\text{O}$}$$ islands grow on Cu-Pt alloy. However, the oxide does not grow deeper into bulk even at high temperature because of less diffusivity of Pt^28^.

### Charge distribution

In Fig. 4, for 0.5 ML-O adsorbed on $$\mbox{$\text{Cu}_{3}\text{Pt}(111)$}$$, we see that in the early stage of oxidation, Cu segregates to the surface and oxidized to form CuO. The charge distribution also shows that the more electronegative Pt competes with O for the Cu electrons. This, together with the mobility arguments presented earlier, accounts for why $$\mbox{$\text{Cu}_{2}\text{O}$}$$ easily forms on Cu(111) and not on Cu-alloys. Note that this could also consistently explain previous reports for that electron transfer from the metal substrate (Au, Ni, Mo, Cu, V) to the metal oxide resulted in $${\text {Mo}}^{6+}$$ reduction to $${\text {Mo}}^{4+}$$ and/or $${\text {Mo}}^{5+}$$ near the interface^41^. Conversely, in our case, electron transfer results in $${\text {Cu}}^{+}$$ oxidation to $${\text {Cu}}^{2+}$$ as shown in Fig. S.11 in supplementary information. Additionally, the presence of single Pt atoms at the Cu metal-oxide interface weakens Cu-O bond^24^, consistent with the preferential formation of CuO (Cu–O: 0.188 and 0.196 nm) than $$\mbox{$\text{Cu}_{2}\text{O}$}$$ (Cu–O: 0.185 nm) on Pt interface^42^.

## Summary and conclusions

In conclusion, we studied the oxidation of $$\mbox{$\text{Cu}_{3}\text{Pd}(111)$}$$ and $$\mbox{$\text{Cu}_{3}\text{Pt}(111)$}$$, using 2.3 eV hyperthermal oxygen molecular beam (HOMB) source, and synchrotron-radiation X-ray photoemission spectroscopy (SR-XPS) for surface characterization. We determined the Pd- and Pt- layer profiles of $$\mbox{$\text{Cu}_{3}\text{Pd}(111)$}$$ and $$\mbox{$\text{Cu}_{3}\text{Pt}(111)$}$$ from the corresponding Pd-3*d* and Pt-4*f* spectra. At $$300\,\text{K}$$, we found mainly (only) the presence of CuO on both $$\mbox{$\text{Cu}_{3}\text{Pd}(111)$}$$ and $$\mbox{$\text{Cu}_{3}\text{Pt}(111)$}$$. At $$500\,\text{K}$$, we found $$\mbox{$\text{Cu}_{2}\text{O}$}$$ on $$\mbox{$\text{Cu}_{3}\text{Pd}(111)$}$$, and only CuO on $$\mbox{$\text{Cu}_{3}\text{Pt}(111)$}$$. For comparison, at $$300\,\text{K}$$, $$\mbox{$\text{Cu}_{2}\text{O}$}$$ forms on Cu(111), and no oxides form on $$\mbox{$\text{Cu}_{3}\text{Au}(111)$}$$. The early formation of Cu oxides on $$\mbox{$\text{Cu}_{3}\text{Pd}(111)$}$$ and $$\mbox{$\text{Cu}_{3}\text{Pt}(111)$}$$ results in hindered reactivity (susceptibility) to further oxidation into the bulk (resulting in the formation of $$\mbox{$\text{Cu}_{2}\text{O}$}$$) as compared to Cu. Cu oxide formation depends on the Cu alloy component and temperature. We ascribe this difference/preference of Cu oxide species to the mobility of the interfacial Cu/Pd/Pt, and the charge transfer between the initial (pre-oxidized) surface (Cu) and subsurface (Cu, Pd, or Pt) species. The presence of $$\mbox{$\text{Cu}_{2}\text{O}$}$$ and metastable CuO at the Pd and Pt interface could play an important role in catalytic reactions. We showed that we can control the oxidation state of the surface metal oxide by alloying, which in turn would allow us to control the catalytic reactivity of the oxides.

## Method

### Experiments

We performed all experiments with the surface reaction analysis apparatus (SUREAC 2000) built at BL23SU in SPring-8^17–20,43,44^, with the base pressure of $$<2\times 10^{-8}$$ Pa. Briefly, our surface reaction analysis chamber has an electron energy analyzer (OMICRON EA125-5MCD) and a Mg/Al-K$$\alpha$$ twin-anode x-ray source (OMICRON DAR400). We also have a quadrupole mass spectrometer, for monitoring the molecular beam, located opposite to the HOMB (hyperthermal oxygen molecular beam) source. We purchased $$\mbox{$\text{Cu}_{3}\text{Pd}(111)$}$$ and $$\mbox{$\text{Cu}_{3}\text{Pt}(111)$}$$ samples from SPL and MaTeck, respectively. We cleaned the $$\mbox{$\text{Cu}_{3}\text{Pd}(111)$}$$ and $$\mbox{$\text{Cu}_{3}\text{Pt}(111)$}$$ samples by repeated sputtering with Ar$$^{+}$$ and annealing for 20 min ($$\mbox{$\text{Cu}_{3}\text{Pd}(111)$}$$: 1.0 keV, $$723\,\text{K}$$, $$\mbox{$\text{Cu}_{3}\text{Pt}(111)$}$$: 0.5 keV, $$773\,\text{K}$$), until the impurities were no longer detectable by SR-XPS (synchrotron-radiation X-ray photoemission spectroscopy). We generated a HOMB by the free expansion of mixed gas of $${\text {O}}_2$$, He and/or Ar from a nozzle with a small orifice. The translational energy of HOMB, $$E_{SG}$$ can be expressed as:

1 $$\begin{aligned} E_{SG}= & {} S^2\cdot R \cdot T_0 \cdot \frac{m_{SG}}{m_{s}}, \end{aligned}$$

where *S* (= 1.557) is a factor that is expressed by using the Mach number, *R* (= $8.617 \times 10^{-5} \, {\text {eV}} \cdot \, {\text {K}}^{-1}$) is gas constant, $T_0$ is the nozzle temperature, $m_{SG}$ is the mass of the reactant gas (${\text {O}}_2$) and $m_s$ is the reduced mass of the mixed gas (He and/or Ar). By changing the gas mixing ratios at the nozzle and nozzle temperature $T_0$, we can control the kinetic energy of the incident HOMB, $E_{SG}$. The detailed explanation for HOMB generation is shown in Refs.^45,46^. We set the nozzle temperature to $1400\,\text{K}$, obtaining a 2.3 eV HOMB. We irradiate the sample surface with a HOMB (along the surface normal) at $T_{\rm S} = 300$ and $500\,\text{K}$. The pressure during the HOMB irradiation is about $1 \times 10^{-5}$ Pa. The oxidation by the scattered ${\text {O}}_2$ which causes the pressure increase is not important because the percentage of ${\text {O}}_2$ in the gas is only 1% for the 2.3 eV HOMB, and the oxidation by thermal ${\text {O}}_2$ is less reactive than by HOMB as shown in Fig. 1. After each irradiation, we then obtained the corresponding high-resolution SR-XPS spectra at $T_{\rm S} = 300\,\text{K}$, at detection angles of $\theta$ = 0$^\circ$ and 70$^\circ$ from the surface normal, using a monochromatic SR beam with a photon energy of 1100 eV. We performed the HOMB irradiations at $T_{\rm S} = 300$ and $500\,\text{K}$.

### Theoretical calculations

We performed density functional theory (DFT)-based total energy calculations as implemented in the Vienna Ab Initio Simulation Package^47,48^, within the generalized gradient approximation (GGA)^49^, using plane waves (600 eV cutoff energy) and the projector augmented wave method^50^. To model $$\mbox{$\text{Cu}_{3}\text{Pd}(111)$}$$ and $$\mbox{$\text{Cu}_{3}\text{Pt}(111)$}$$, we used slabs. Each slab has seven fcc(111) layers, separated by ca. 1.50 nm ($$\mbox{$\text{Cu}_{3}\text{Pd}$}$$) and 0.7 nm ($$\mbox{$\text{Cu}_{3}\text{Pt}$}$$) vacuum, repeated in a supercell geometry (shown in Fig. S.2). We also applied dipole corrections. Each layer in the slab contains 4 atoms, so that the composition (of Pd or Pt) can be varied in steps of $$25\%$$. Convergence tests for *k*-point meshes and cutoff energy values were performed (cf., Table. S.1). We have chosen sufficiently large supercells so as to avoid interaction between adsorbates in the neighboring supercells. We performed Brillouin zone integration using the Monkhorst-Pack special *k*-point sampling technique^51^, with $$8 \times 8 \times 1$$ ($$\mbox{$\text{Cu}_{3}\text{Pd}$}$$) and $$6 \times 6 \times 1$$ ($$\mbox{$\text{Cu}_{3}\text{Pt}$}$$) sampling meshes. We kept the bottom five (four) layers of $$\mbox{$\text{Cu}_{3}\text{Pd}$}$$ ($$\mbox{$\text{Cu}_{3}\text{Pt}$}$$), which comprise the unsegregated layers having bulk stoichiometry, viz., $$25\%$$-Pd (or Pt) $$75\%$$-Cu in the $${\text {L1}}_{2}$$ ordered structure, fixed to the optimized theoretical bulk lattice constant 0.3723 nm (0.3730 nm). We allowed the top three layers, which constitute the segregated layers, viz., the first surface layer, the second-, and third-(sub-surface) layers, to relax. In addition to calculations for the slab, we also carried out similar calculations for bulk Cu, bulk Pd, bulk Pt, bulk $$\mbox{$\text{Cu}_{3}\text{Pd}$}$$ and bulk $$\mbox{$\text{Cu}_{3}\text{Pt}$}$$.

## Supplementary information

Supplementary Information.

## Data Availability

All data needed to evaluate the conclusions in the paper are present in the paper and/or the Supplementary Information. Additional data related to this paper may be requested from the authors.
