# Supplementary material for: Interface atom mobility and charge transfer effects on CuO and Cu2O formation on Cu3Pd(111) and Cu3Pt(111)
Source: Sci Rep. 2021 Feb 15;11:3906. doi: 10.1038/s41598-021-82180-w (PMC7884792; doi:10.1038/s41598-021-82180-w)
Supplement: Supplementary file 1 — Supplementary Information. [file 41598_2021_82180_MOESM1_ESM.pdf]

Supplementary information for:

Interface Atom Mobility and Charge Transfer Effects on CuO and Cu_2O Formation on $Cu_3Pd(111)$ and $Cu_3Pt(111)$

*Yasutaka Tsuda,[1,2] Jessiel Siaron Gueriba,[3,4] Takamasa Makino,[1]
†Wilson Agerico Diño,[3,5] Akitaka Yoshigoe,[2] and ‡Michio Okada[1,6]

[1] *Department of Chemistry, Osaka University, Toyonaka, Osaka 560-0043, Japan*
[2] *Materials Sciences Research Center, Japan Atomic Energy Agency, 1-1-1 Kouto, Sayo-cho, Sayo-gun, Hyogo 679-5148, Japan*
[3] *Department of Applied Physics, Osaka University, Suita, Osaka 565-0871, Japan*
[4] *Department of Physics, De La Salle University, 2401 Taft Avenue, Manila, 0922, Philippines*
[5] *Center for Atomic and Molecular Technologies, Osaka University, Suita, Osaka 565-0871, Japan*
[6] *Institute for Radiation Sciences, Osaka University, Toyonaka, Osaka 560-0043, Japan*
* *tsuda.yasutaka@jaea.go.jp*
† *wilson@dyn.ap.eng.osaka-u.ac.jp*
‡ *okada@chem.sci.osaka-u.ac.jp*

Contents

S.1. Layer profiles from the clean Pd-3*d* and Pt-4*f* spectra 2

S.2. Layer profiles from DFT calculation 4

S.3. LEED analysis for clean $Cu_3Pd(111)$ surface 6

S.4. Oxygen coverage dependence of the Pd-3*d* and Pt-4*f* spectra 7

S.5. Peak shape analyses of the O-1*s* spectra 8

S.6. Valence band spectra of clean Cu(111), $Cu_3Au(111)$, $Cu_3Pd(111)$, and $Cu_3Pt(111)$ 12

S.7. Schematic Energy Band Diagram 13

References 14

S.1. Layer profiles from the clean Pd-3d and Pt-4f spectra

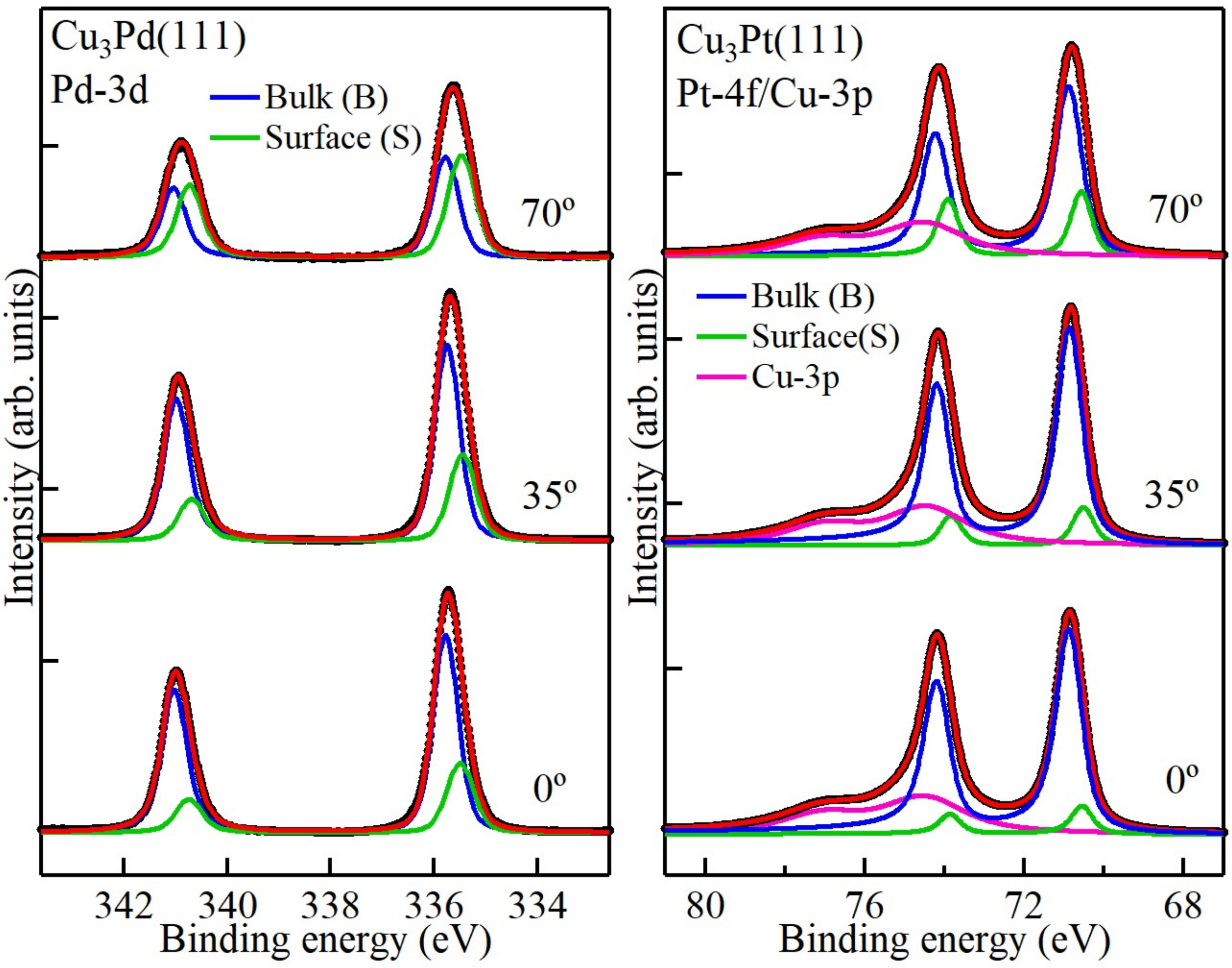

Fig. S.1. Detection angle dependence of Pd-3d and Pt-4f XPS spectra on clean $Cu_3Pd(111)$ (left panel) and $Cu_3Pt(111)$ (right panel), respectively, taken at photoelectron detection angles $\theta = 0°, 35°, 70°$ from the surface normal. The XPS spectra can be clearly separated into a bulk (B-) component (blue line) and a surface (S-) component (green line). Cu-3p components overlap with Pt-4f on $Cu_3Pt(111)$. Background subtracted using the Shirley method [S1]. Intensities given in arbitrary units and scales vary across panels (i.e., differ between samples and detection angles).

In Figure S.1, we show the Pd-3d and Pt-4f SR-XPS spectra from clean (0.00 ML O-coverage) $Cu_3Pd(111)$ (left panel) and $Cu_3Pt(111)$ (right panel), respectively, taken at photoelectron detection angles $\theta = 0°, 35°, 70°$ from surface normal. Note that on Cu_3Pt, the Pt-4f spectra overlap with the Cu-3p spectra. We fit the spectra with the Voigt function and subtract the background using the Shirley method [S1]. We can separate both the Pd-3d and Pt-4f spectra into bulk (B-: from high binding energy bulk photolectrons) and surface (S-: from low binding energy surface photoelectrons) components (cf., blue and green curves in Figure S.1). The $Cu_3Pd(111)$ B-components appear at 335.7 eV (Pd-3$d_{5/2}$) and 340.9 eV (Pd-3$d_{3/2}$), as compared to previous reports of Pd-3$d_{5/2}$ peak position at 334.9 eV [S2]. We may ascribe the slight difference to alloying with Cu [S3]. The $Cu_3Pt(111)$ B-components appears at 70.9 eV (Pt-4$f_{7/2}$) and 74.2 eV (Pt-4$f_{5/2}$). Earlier reports Pt-4$f_{7/2}$ peak positions at 71.2 eV [S4] and B-component at 70.9 eV [S5, S6]. Again the slight shifts due to alloying with Cu. We observe surface core-level shift (SCLS) values of -280 meV on $Cu_3Pd(111)$ and -327 meV on $Cu_3Pt(111)$, which can be compared with previous reported values of -0.3 eV [S2] and -0.36 ± 0.02 eV [S6], respectively.

From the photoelectron detection angle dependence of the B- and S-component intensities, we can then determine

the Pd and Pt layer concentration profiles on the alloy surfaces. First, we approximate the S to B peak intensity ratio ($\frac{A_S}{A_B}$) using the following equation:

$$\begin{aligned} \frac{A_S}{A_B} &= \frac{x_1}{\sum_{n=2}^{\infty} x_n \exp\left(-\frac{(n-1)d}{\lambda \cos\theta}\right)} \\ &= \frac{x_1}{x_2 \exp\left(-\frac{d}{\lambda \cos\theta}\right) + \sum_{n=2}^{\infty} x_n \exp\left(-\frac{(n-1)d}{\lambda \cos\theta}\right)}. \end{aligned} \tag{1}$$

x_n gives the Pd or Pt fraction (%-Pd or %-Pt) of the n-th layer from the surface, d the interlayer distance, and θ the photoelectron detection angle from the surface normal. For a Pd-3d electron in Cu_3Pd, we obtain a photoelectron mean free path $\lambda = 1.27$ nm, following previously reported procedures [S10]. Similarly, for a Pt-4f electron in Cu_3Pt, $\lambda = 1.49$ nm. Note the photon energy 1100 eV in our experimental condition. On $Cu_3Pd(111)$, assuming bulk concentrations for layers $n \geq 3$, i.e., $x_{n\geq 3} = 25$, we find $x_1 = 22$ and $x_2 = 44$, using two equations obtained by considering $\theta = 0°$ and 70°. On $Cu_3Pt(\bar{1}11)$, we cannot obtain the proper values of x_n ($0 \leq x_n \leq 1$) by assuming bulk concentrations for layers $n \geq 3$, i.e., $x_{n\geq 3} = 25$. Instead, we assumed $x_{n\geq 5} = 25$ and found $x_1 = 23$, $x_2 = 33$, $x_3 = 88$, and $x_4 = 20$, using four equations considering $\theta = 0°, 35°, 60°, 70°$.

S.2. Layer profiles from DFT calculation

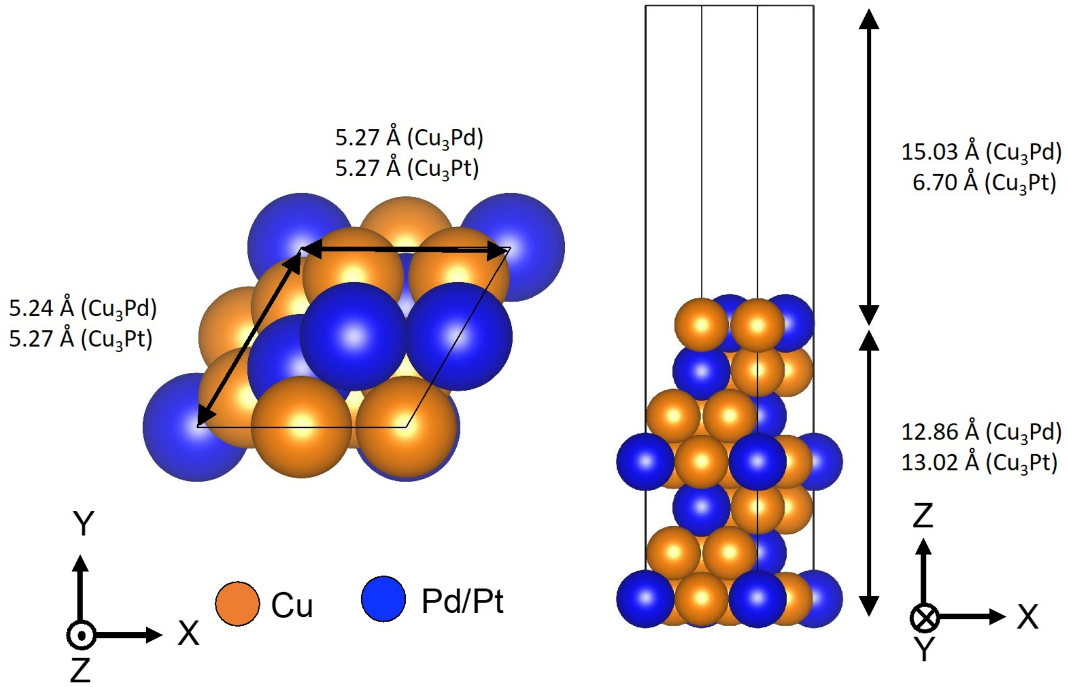

Fig. S.2. The seven-layer slab model used in the calculations for $Cu_3Pd(111)$ and $Cu_3Pt(111)$. The orange balls and blue balls correspond to Cu atoms and Pd(Pt) atoms, respectively. We show the representative structure of $(x_1, x_2.x_3, x_{bulk})$ = (0.50, 0.25, 0.25, 0.25)

Table S.1. Convergence test for k-point meshes and cutoff energy values.

Cu_3Pd (111)				Cu_3Pt (111)			
K-point	Total Energy (eV)	Cutoff Energy (eV)	Total Energy (eV)	K-point	Total Energy (eV)	Cutoff Energy (eV)	Total Energy (eV)
4 x 4 x 1	-46.4966	400	-46.5278	4 x 4 x 1	-62.5668	400	-62.4942
5 x 5 x 1	-46.5551	450	-46.5203	5 x 5 x 1	-62.4126	450	-62.498
6 x 6 x 1	-46.5201	500	-46.5201	6 x 6 x 1	-62.4954	500	-62.4954
7 x 7 x 1	-46.5593	550	-46.5248	7 x 7 x 1	-62.4932	550	-62.4958
8 x 8 x 1	-46.5546	600	-46.5206	8 x 8 x 1	-62.4992	600	-62.4941
9 x 9 x 1	-46.4946	650	-46.5211	9 x 9 x 1	-62.5107	650	-62.4939
10 x 10 x 1	-46.5547	700	-46.5249	10 x 10 x 1	-62.4991	700	-62.5043

Following some detailed thermodynamic derivations in the literature (cf., e.g., Refs. [S10, S11, S12, S13]), we determine the surface segregation of clean $Cu_3Pd(111)$ and $Cu_3Pt(111)$ using the slab model to calculate the corresponding surface free energy γ:

$$\gamma = \frac{1}{A}\left\{G_{\rm slab} - \sum_M \mu_M N_M\right\}. \tag{2}$$

A gives the surface area of the slab, $G_{\rm slab}$ the Gibbs free energy of the slab, μ_M the chemical potentials of each atomic species M $(= \mathrm{Cu}, \mathrm{Pd}, \mathrm{Pt})$, and N_M the number of each atomic species M in the slab. Assuming equilibrium conditions (between alloy surface and the underlying bulk reservoir), we have

$$\mu_{\rm bulk} = x_{M'}^{\rm bulk} \Delta\mu_{{\rm Cu}-M'} + \mu_{\rm Cu}, \tag{3}$$

where

$$\Delta\mu_{\mathrm{Cu}-M'} = \mu_{M'} - \mu_{\mathrm{Cu}}, \tag{4}$$

and $x_{M'}^{\mathrm{bulk}}$ gives the mole fraction of the atomic species M' $(= \mathrm{Pd}, \mathrm{Pt})$. As an estimate, $\Delta\mu_{\mathrm{Cu}-M'}$ takes values ranging from that corresponding to the phase separation of Cu (Cu-rich limit, when the bulk reservoir is rich in Cu), to that corresponding to the phase separation of M' (M'-rich limit, when the bulk reservoir is rich in M'). From Eq. (4)

$$\mu_{\mathrm{Cu}-M'}^{M'-\mathrm{rich}} \leq \Delta\mu_{\mathrm{Cu}-M'} \leq \mu_{\mathrm{Cu}-M'}^{\mathrm{Cu}-\mathrm{rich}}, \tag{5}$$

where

$$\mu_{\mathrm{Cu}-M'}^{M'-\mathrm{rich}} = \frac{\mu_{M'}^{\mathrm{fcc}} - \mu_{\mathrm{Cu}_3M'}^{\mathrm{bulk}}}{1 - x_{M'}^{\mathrm{bulk}}} = \frac{\mu_{M'}^{\mathrm{fcc}} - \mu_{\mathrm{Cu}_3M'}^{\mathrm{bulk}}}{0.75} \tag{6}$$

and

$$\mu_{\mathrm{Cu}-M'}^{\mathrm{Cu}-\mathrm{rich}} = \frac{\mu_{\mathrm{Cu}_3M'}^{\mathrm{bulk}} - \mu_{\mathrm{Cu}}^{\mathrm{fcc}}}{1 - x_{\mathrm{Cu}}^{\mathrm{bulk}}} = \frac{\mu_{\mathrm{Cu}_3M'}^{\mathrm{bulk}} - \mu_{\mathrm{Cu}}^{\mathrm{fcc}}}{0.25}. \tag{7}$$

$\mu_{\mathrm{Cu}}^{\mathrm{fcc}}$ and $\mu_{M'}^{\mathrm{fcc}}$ give the chemical potentials of Cu and M' in the fcc bulk, respectively. We show the calculated surface energies in Figure S.3. To estimate $\Delta\mu_{\mathrm{Cu}-M'}$ for Cu_3M' ($\Delta\mu_{\mathrm{Cu}_3M'}$), we took the difference in energy for two arbitrarily large cells made up of $N_{\mathrm{Cu}} + N_{M'} = 101$ atoms, with corresponding compositions ($N_{\mathrm{Cu}} = 75, N_{M'} = 25$, with one Cu) and ($N_{\mathrm{Cu}} = 76, N_{M'} = 24$, and one more M'). In Figure S.3, we show the condition that gives the smallest γ at $\Delta\mu_{\mathrm{Cu}_3M'}$ as a red line.

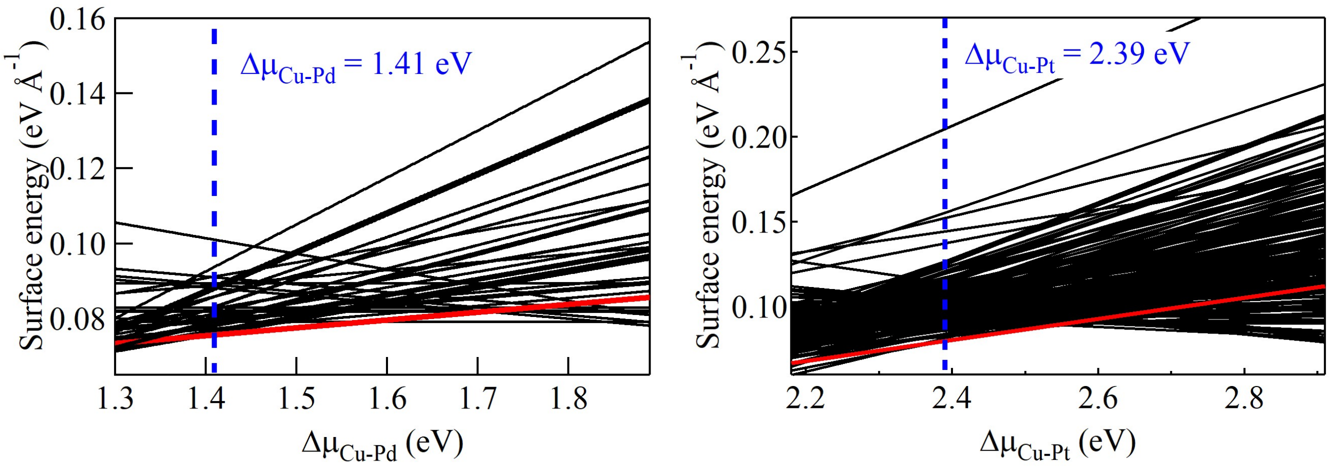

Fig. S.3. Calculated surface free energies for $\mathrm{Cu_3Pd}(111)$ and $\mathrm{Cu_3Pt}(111)$, as a function of the chemical potential difference $\Delta\mu_{\mathrm{Cu}-M'} = \mu_{\mathrm{Cu}} - \mu_{M'}$ between Cu and $M'(= \mathrm{Pd}, \mathrm{Pt})$. Red lines indicate the ground state structure for $\Delta\mu_{\mathrm{Cu-Pd}} = 1.41$ eV (broken vertical blue line, left panel) and $\Delta\mu_{\mathrm{Cu-Pt}} = 2.39$ eV (broken vertical blue line, right panel).

S.3. LEED analysis for clean $Cu_3Pd(111)$ surface

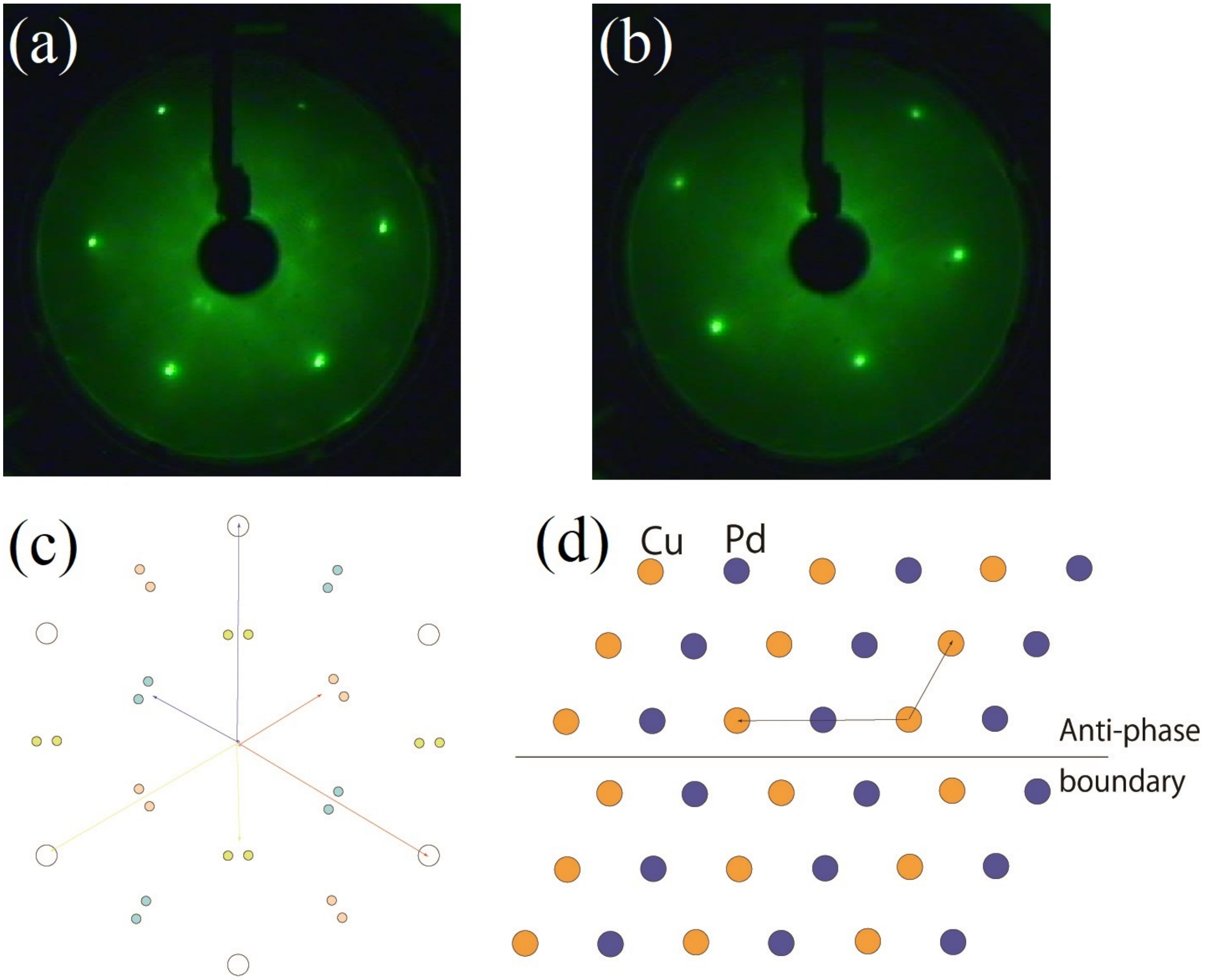

Fig. S.4. (a) LEED image for clean $Cu_3Pd(111)$, obtained with an electron energy of 103.60 eV. (b) LEED image for clean $Cu_3Pt(111)$, obtained with an electron energy of 103.86 eV. (c) Schematic depiction of the three rotationally symmetric domains. (d) (2×1) $Cu_3Pd(111)$ structure with domain boundary.

S.4. Oxygen coverage dependence of the Pd-3d and Pt-4f spectra

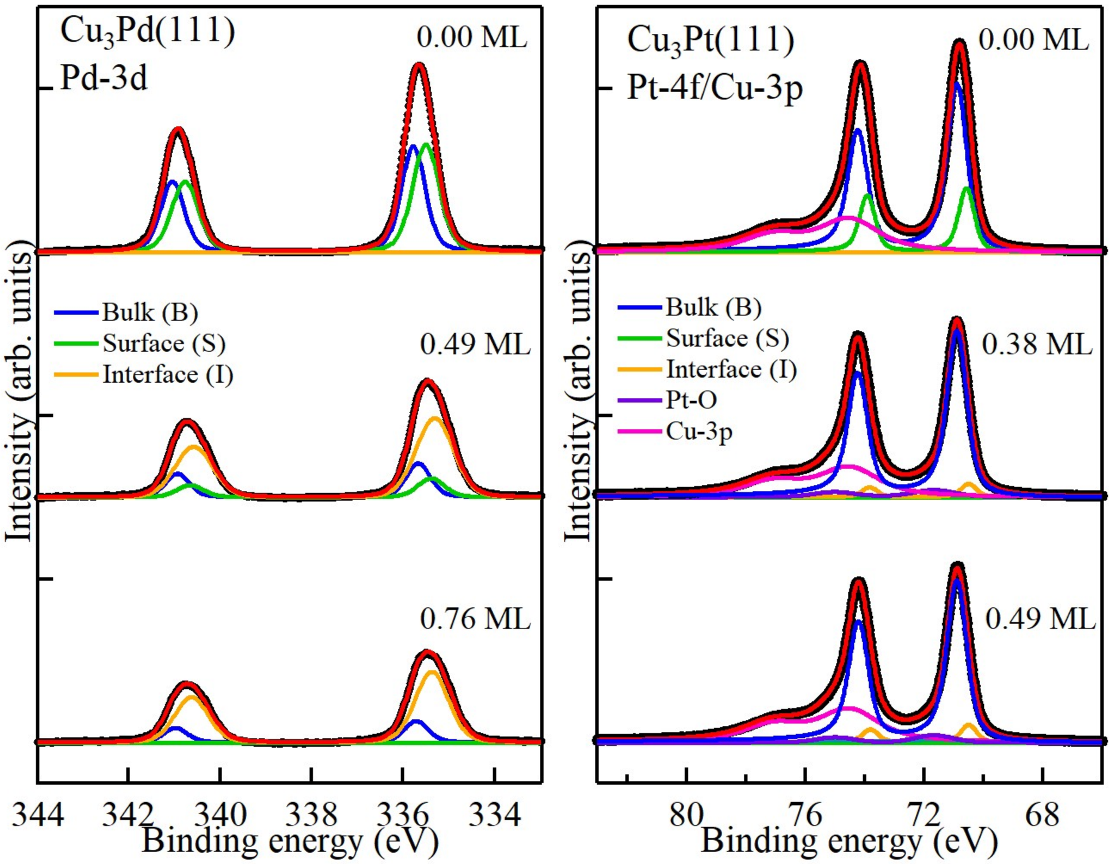

Fig. S.5. Oxygen coverage dependence of Pd-3d and Pt-4f XPS spectra on clean $Cu_3Pd(111)$ and $Cu_3Pt(111)$, respectively, at a photoelectron detection angle $\theta = 70°$ from the surface normal. The XPS spectra can be clearly separated into a bulk (B) component (blue line), a surface (S) component (green line), and an interface-layer (I) component (yellow line). The Pt-4f contains a small Pt-O component. Cu-3p component is overlapped with Pt-4f on $Cu_3Pt(111)$. Background subtracted by the Shirley method [S1]. Intensities given in arbitrary units and scales vary across panels (i.e., differ between samples and O-coverages).

In Figure S.5, we show the oxygen coverage dependence of the Pd-3d and Pt-4f SR-XPS spectra of oxidized $Cu_3Pd(111)$ and $Cu_3Pt(111)$, after 2.3 eV HOMB irradiation at 300 K, respectively, taken at a photoelectron detection angle $\theta = 0°$ from the surface normal. We can separate the Pd-3d and Pt-4f spectra into three peaks, viz., bulk (B-), surface (S-), and interface (I-) components. The I-component develops at CLS = −360 meV on the $Cu_3Pd(111)$ and CLS = −370 meV on the $Cu_3Pt(111)$, with increasing O-coverage. No peak growth observed at binding energies higher than that of the Pd-3d spectra B-component. This indicates absence of surface Pd oxides, i.e., only Cu oxides exist on the surface. Therefore, we assigned the Pd-3d spectra I-component to the interface Pd (located between the bulk and surface Cu oxide layer). On the other hand, we find a broadening small peak growing at binding energies higher than that of the Pt-4f spectra B-component (CLS $\sim$ +0.8 eV). From previous reports [S4, S5, S6], we cannot assign this component to Pt oxides nor to Pt under the adsorbed O atoms. This must indicate interface Pt interacting with O atoms. Due to higher diffusion barrier of Pt, this Pt-O can be formed through the CIA process (cf., Section on *Mobility/Diffusion* in the main text) at 300 K, while Pd-O cannot. Because the Gibbs energy of formation of both PdO (ca. −190 kJ mol^{-1}) and PtO (ca. −90 kJ mol^{-1}) are lower than that of CuO (ca. −230 kJ mol^{-1}) and Cu_2O (ca. −293 kJ mol^{-1}) at 300 K [S7, S8, S9], this Pt-O is a metastable state. The Pt-O component does not remain on Pt-4f at 500 K (not shown). This indicates that metastable Pt-O cannot be formed because of enhanced diffusion at 500 K. As this component is so small that the corresponding component does not appear on the O-1s spectra (Figure S.7), it again indicates predominantly Cu oxidation on $Cu_3Pt(111)$. We can also assign the I-component to the interface Pt between bulk and surface Cu oxide.

S.5. Peak shape analyses of the O-1s spectra

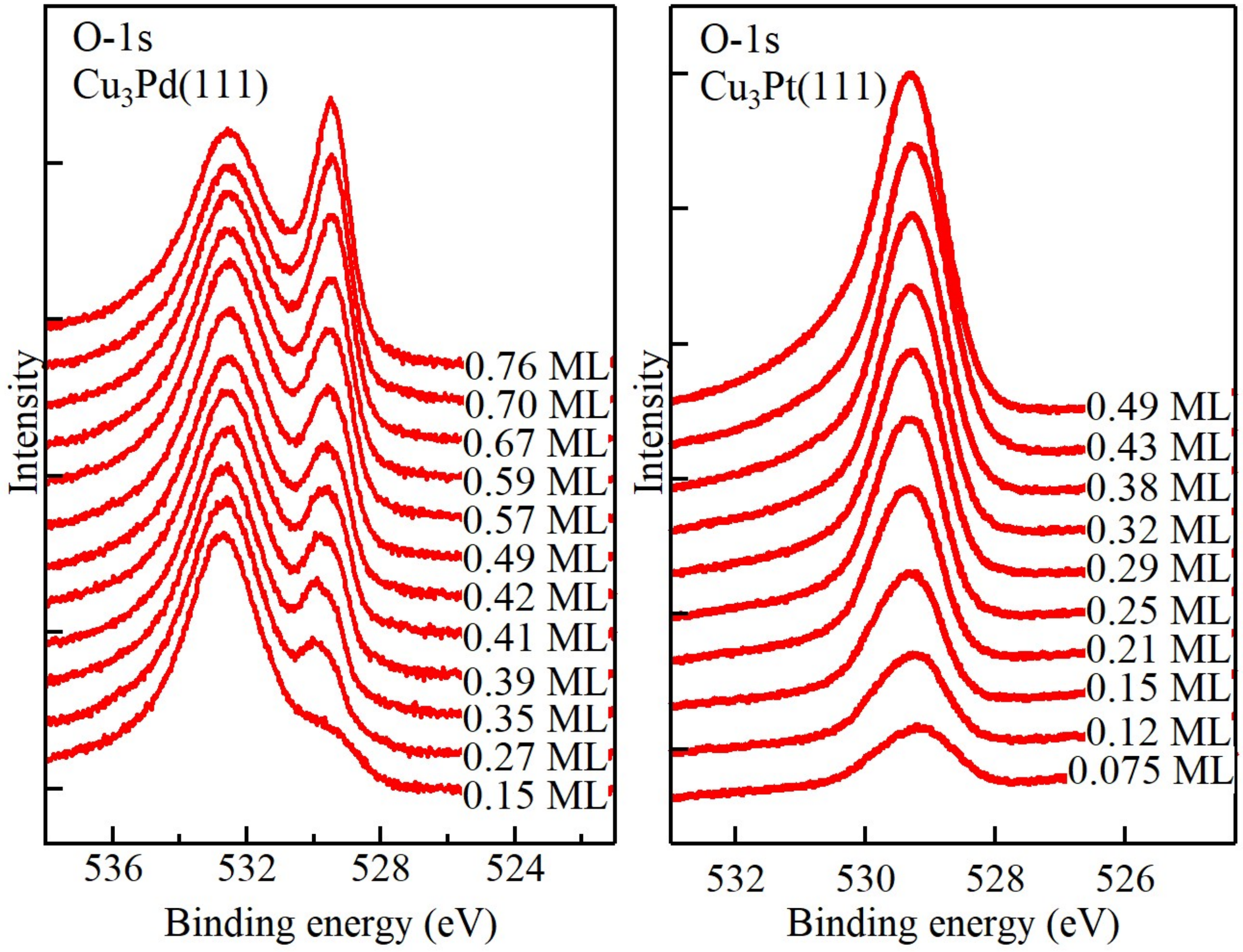

Fig. S.6. O coverage dependence of O-1s XPS spectra on clean $Cu_3Pd(111)$ and $Cu_3Pt(111)$, respectively. The photoelectron detection angle $\theta = 0^\circ$ from the surface normal.

In Figure S.6, we show the O-1s XPS spectra on the $Cu_3Pd(111)$ and $Cu_3Pt(111)$, oxidized at 300 K by 2.3 eV HOMB irradation. We fitted each O-1s XPS peak as shown in Figure S.7. Background subtracted by the Shirley method [S1]. The O-1s spectra on $Cu_3Pd(111)$ and $Cu_3Pt(111)$ contain three components, corresponding to the O atoms adsorbed on surface Cu (529.8 eV), O atoms from CuO(529.2 eV), and O atoms from from Cu_2O (530.2 eV[S14, S15, S16]. On $Cu_3Pd(111)$, O-1s spectra overlap with Pd-3p. On $Cu_3Pt(111)$, we needed one more component to fit the O-1s spectra, i.e., corresponding to the O atoms on CuO (@531.1 eV) [S17]. Integrating each O-1s spectra component measured after 2.3 eV HOMB irradiation at 300 K and 500 K, we can obtain the uptake curves in Figure S.8. For reference, we also plot the area intensities Cu-2p spectra of the CuO satellites (cf., black diamonds in Figure S.8). We can verify that the validity of the O-1s peak fitting from the overlap of CuO satellite intensities with the uptake curves of CuO components. In Fig. S.9, we show the effect of annealing on the corresponding Cu-1s spectra of $Cu_3Pd(111)$ and $Cu_3Pt(111)$ oxidized at $T_S = 300$ K. We can see the decrease in the CuO components and increase in the Cu_2O components at high surface temperature. The Cu_2O component on $Cu_3Pt(111)$ shifts to lower binding energy after annealing. Pt interface layer may cause this by inducing charge transfer (cf., Section on *Charge distribution* in the main text and S.7). At 300 K, Cu_2O can forms near Pt interface as an metastable state by CIA process, The Cu_2O under this condition can be positively (+) charged. Annealing can cause oxidation of Cu_2O to CuO near Pt interface, while the net amount of Cu_2O increases, resulting in the peak shift of Cu_2O component.

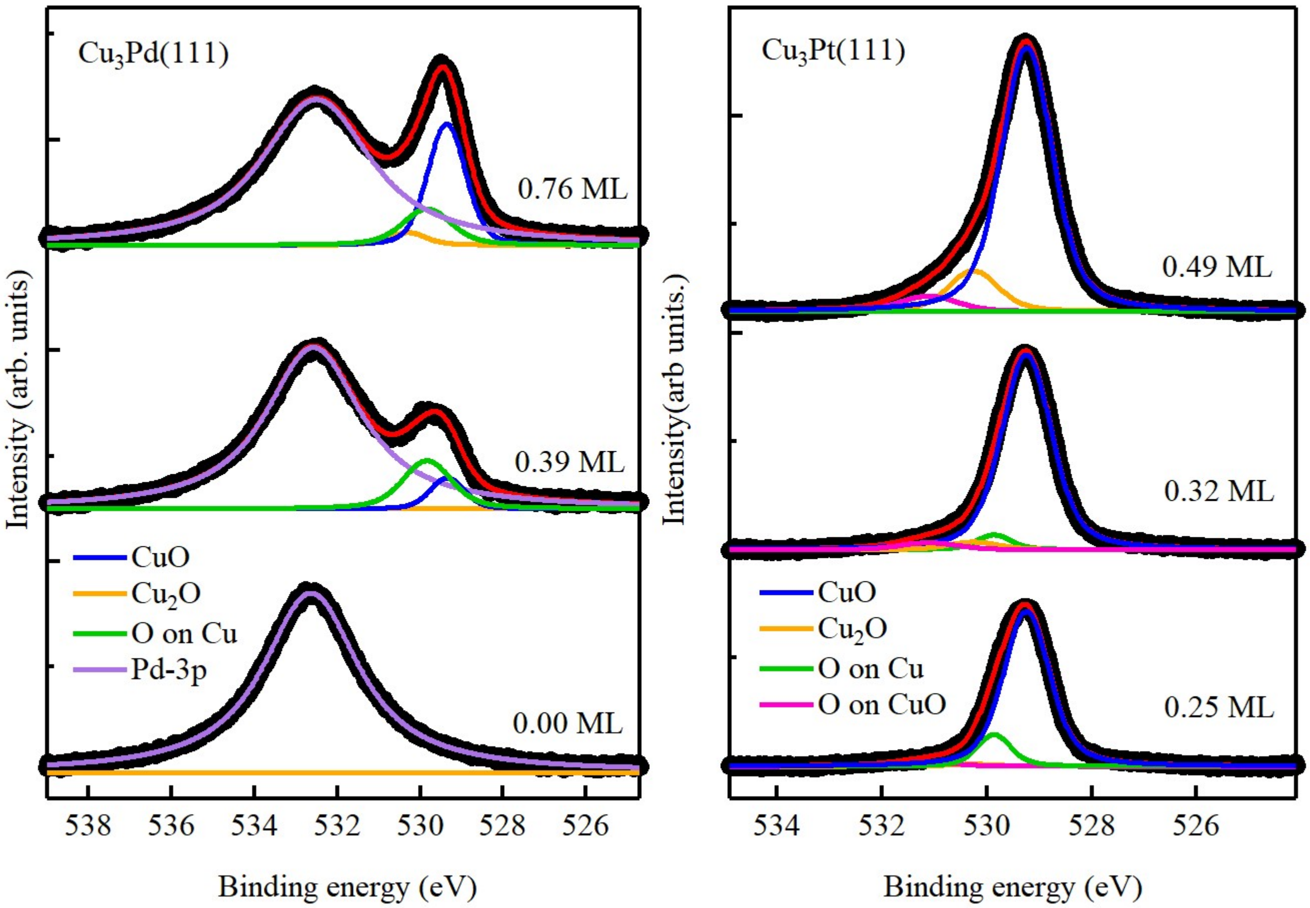

Fig. S.7. Peak shape analyses of the O-1s spectra measured for the $Cu_3Pd(111)$ and $Cu_3Pt(111)$ surface, oxidized at 300 K. Background subtracted by the Shirley method [S1]. The components correspond to the adsorbed O atoms (green line), Cu_2O (orange line) and CuO (blue line). The O-1s peak on $Cu_3Pd(111)$ overlaps with Pd-3p. The O-1s peak on $Cu_3Pt(111)$ contains a small component, corresponding to the O atoms adsorbed on CuO. Intensities given in arbitrary units and scales vary across panels (i.e., differ between samples and O-coverages).

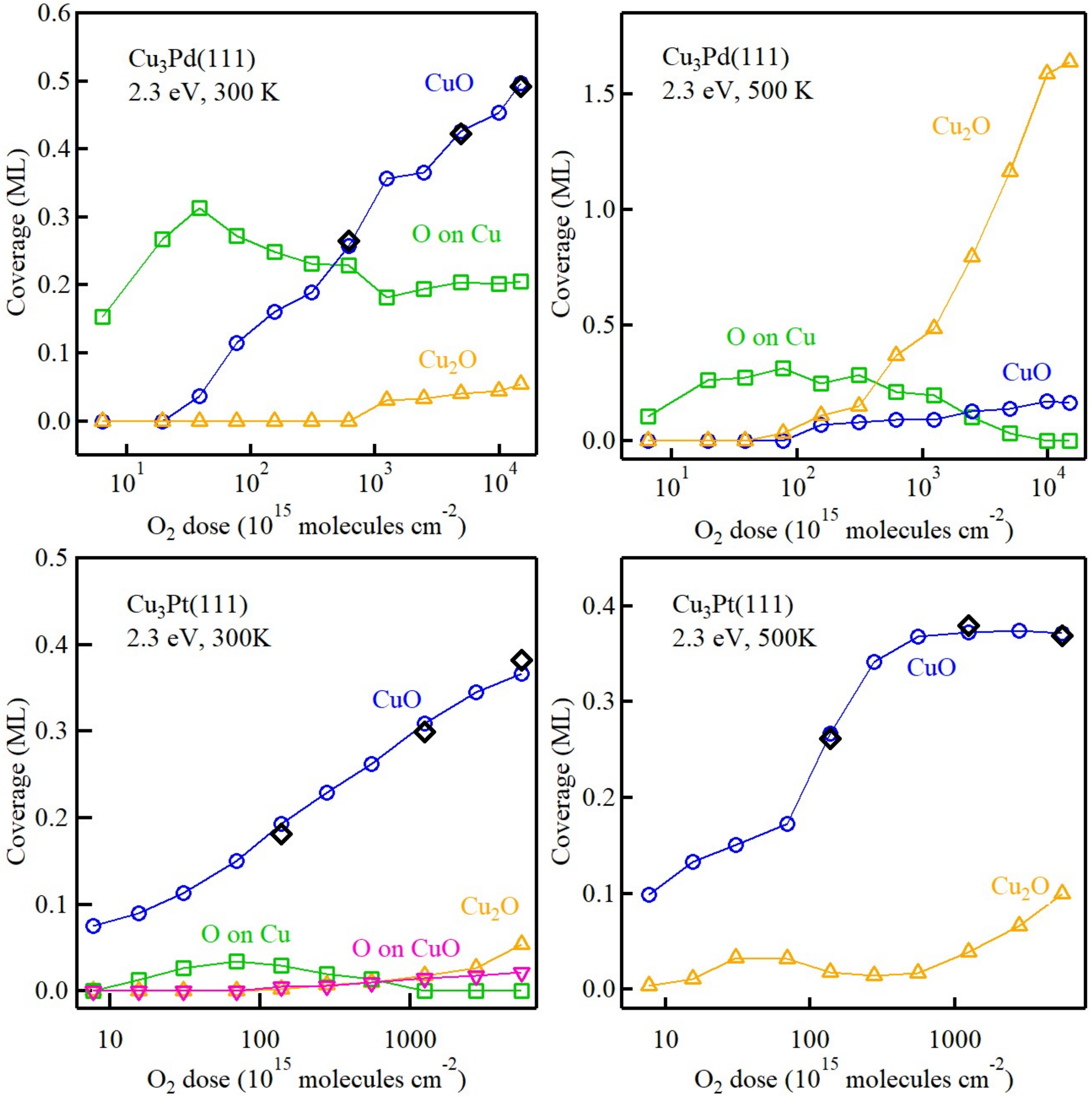

Fig. S.8. O uptake curves of the separated O-1*s* components for the $Cu_3Pd(111)$ and $Cu_3Pt(111)$ surface oxidized by 2.3 eV HOMB irradiation at 300 K and 500 K. Area intensities of the CuO satellites on Cu-2*p* spectra also plotted for $Cu_3Pd(111)$ at 300 K and $Cu_3Pt(111)$ at 300 K and 500 K (black diamonds).

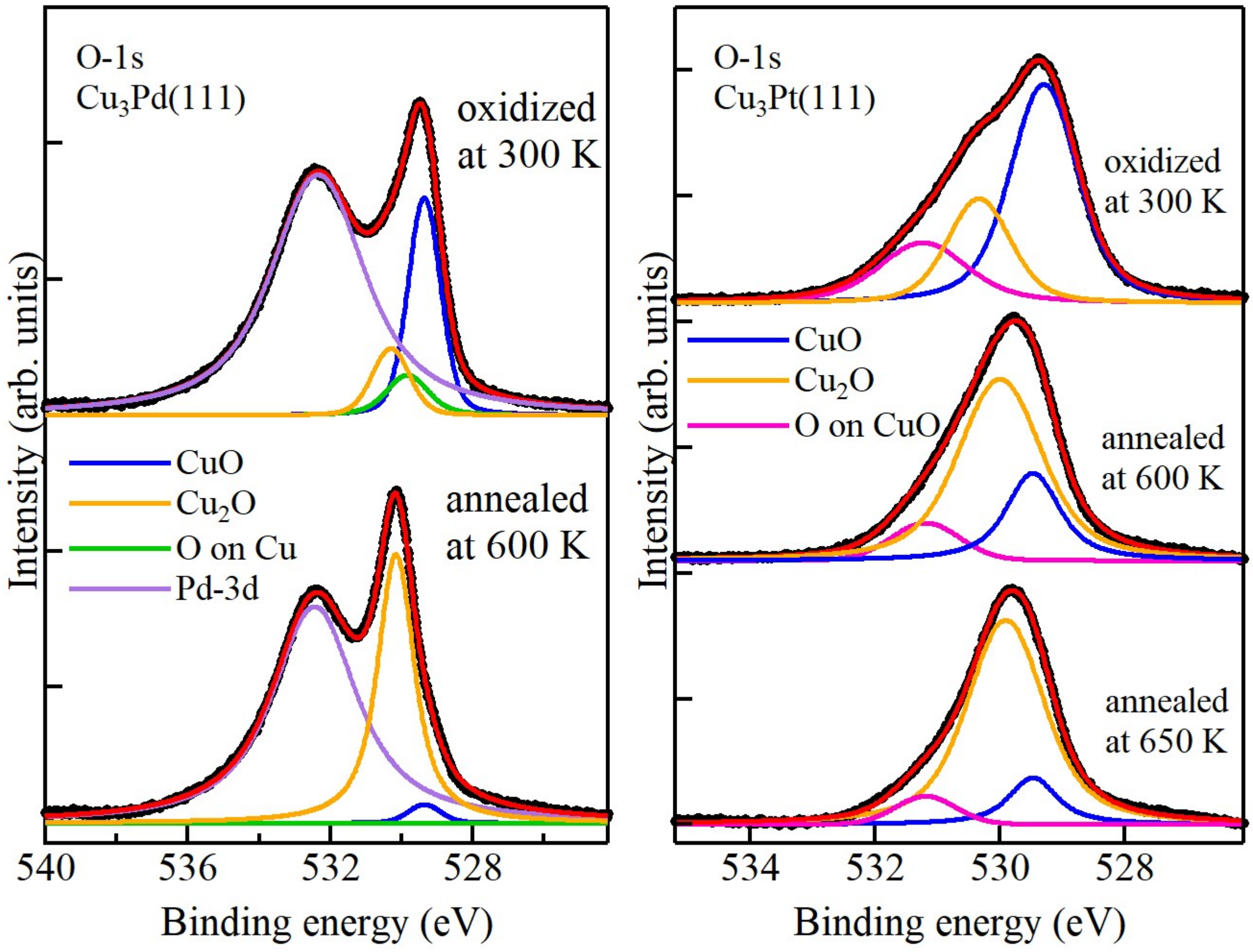

Fig. S.9. Change in O-1*s* XPS spectra on $Cu_3Pd(111)$ and $Cu_3Pt(111)$ after annealing.

S.6. Valence band spectra of clean Cu(111), $Cu_3Au(111)$, $Cu_3Pd(111)$, and $Cu_3Pt(111)$

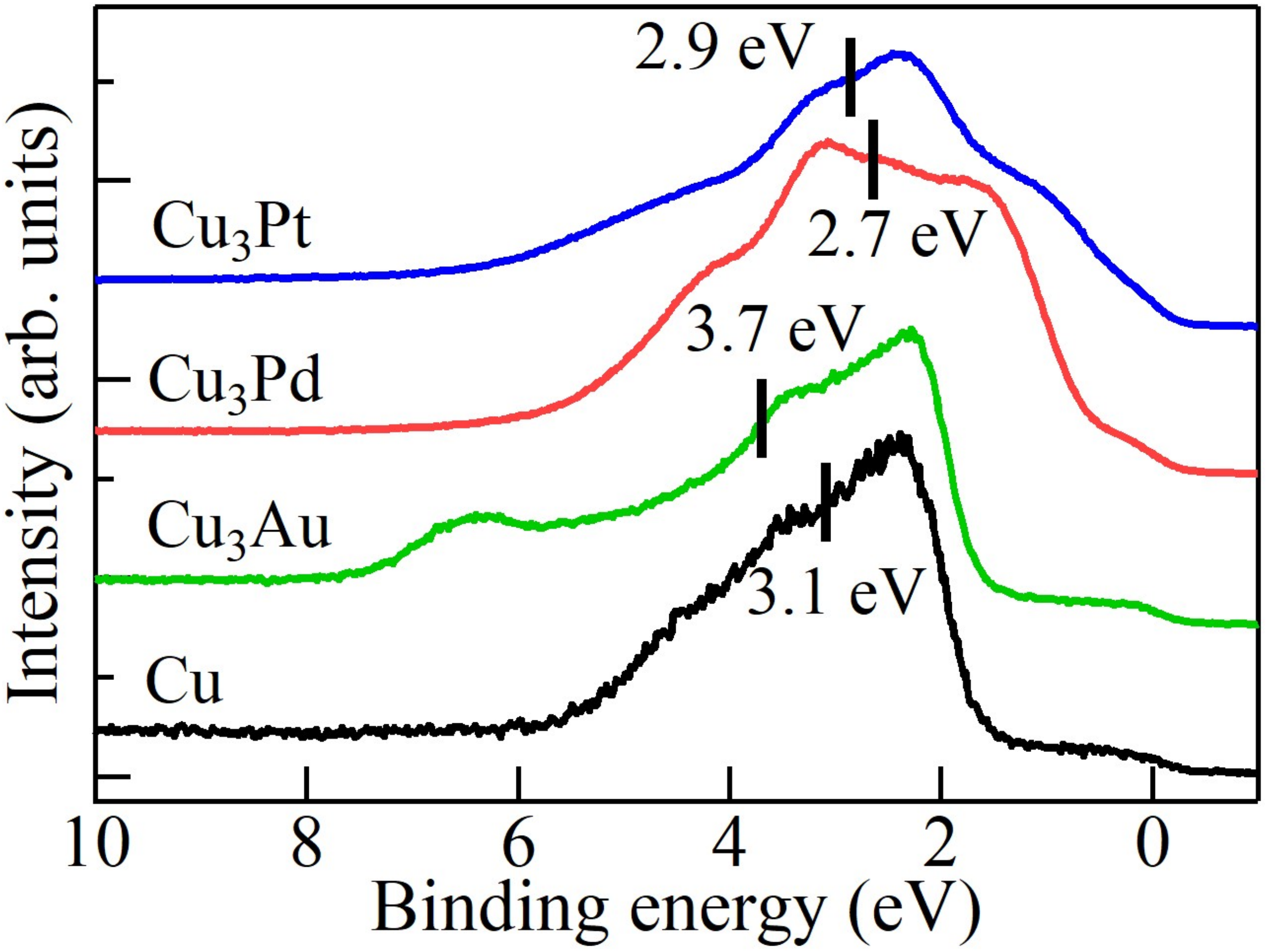

Fig. S.10. Valence band spectra on clean Cu(111), $Cu_3Au(111)$, $Cu_3Pd(111)$, and $Cu_3Pt(111)$, taken at photoelectron detection angle $\theta = 0^\circ$ from the surface normal.

S.7. Schematic Energy Band Diagram

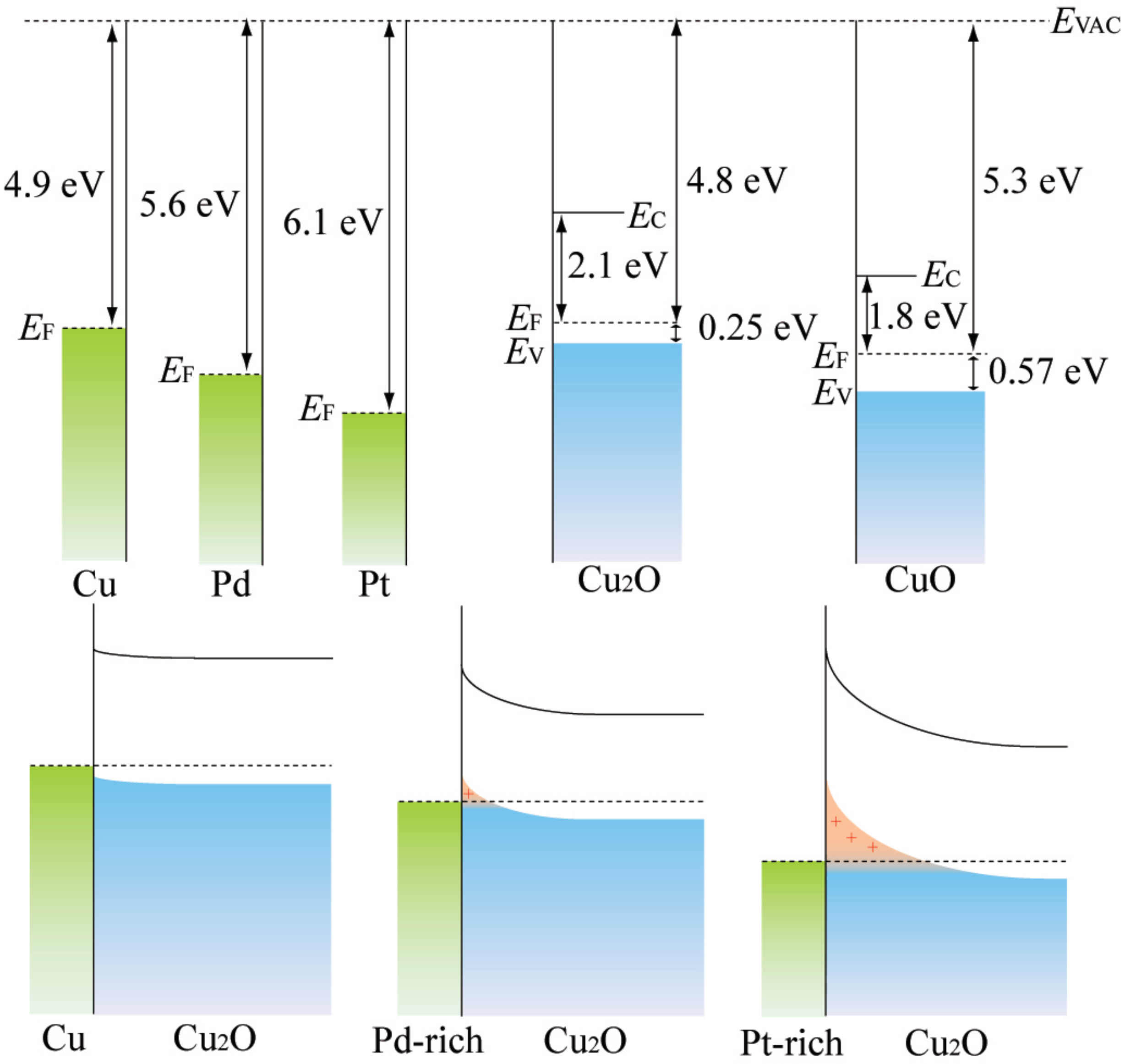

Fig. S.11. (upper panel) Schematic energy band diagrams of metallic Cu, Pd, and Pt, and semiconducting/insulating Cu_2O and CuO. E_F: corresponding Fermi level, E_V: top of the valence band, and E_C: bottom of the conduction band are shown with respect to E_{VAC}: vacuum level. (lower panel) Depiction of the band bending/charge transfer at the metal-semiconductor interface. For the work function of the Pd-rich layer, we take 5.6 eV (that of pure Pd(111) [S18]). For the Pt-rich layers, we take 6.1 eV (that of pure Pt(111) [S18, S19]). We then assume that the work functions of binary alloys (often) monotonically increase or decrease with alloy composition [S23, S24, S25, S26]. Cu_2O has a work function of 4.8 eV [S20, S21] and CuO has 5.3 eV [S22].

References

[S1] Shirley, D. A. High-Resolution X-Ray Photoemission Spectrum of the Valence Bands of Gold. *Phys. Rev. B* **5**, 4709–4714 (1972).

[S2] Leisenberger, F. *et al.* Surface and subsurface oxygen on Pd(111). *Surf. Sci.* **445**, 380 – 393 (2000).

[S3] Mårtensson, N., Nyholm, R., Calén, H., Hedman, J. & Johansson, B. Electron-spectroscopic studies of the Cu_xPd_{1-x} alloy system: Chemical-shift effects and valence-electron spectra. *Phys. Rev. B* **24**, 1725–1738 (1981).

[S4] Peuckert, M. & Bonzel, H. Characterization of oxidized platinum surfaces by X-ray photoelectron spectroscopy. *Surf. Sci.* **145**, 239 – 259 (1984).

[S5] Puglia, C. *et al.* Physisorbed, chemisorbed and dissociated O_2 on Pt(111) studied by different core level spectroscopy methods. *Surf. Sci.* **342**, 119 – 133 (1995).

[S6] Píš, I., Magnano, E., Nappini, S. & Bondino, F. Under-cover stabilization and reactivity of a dense carbon monoxide layer on Pt(111). *Chem. Sci.* **10**, 1857–1865 (2019).

[S7] Balart, M. J., Patel, J. B., Gao, F. & Fan, Z. Grain Refinement of Deoxidized Copper. *Metall. Mater. Trans. A* **47**, 4988–5011 (2016).

[S8] Leparmentier, S., Auguste, J.-L., Humbert, G., Delaizir, G. & Delepine-Lesoille, S. Fabrication of optical fibers with palladium metallic particles embedded into the silica cladding. *Opt. Mater. Express* **5**, 2578–2586 (2015).

[S9] McConnell, M. S. *et al.* Atomic layer deposition of Al_2O_3 for single electron transistors utilizing Pt oxidation and reduction. *J. Vac. Sci. Technol. A* **34**, 01A139 (2016).

[S10] Seah, M. P. & Dench, W. A. Quantitative electron spectroscopy of surfaces: A standard data base for electron inelastic mean free paths in solids. *Surf. Interface Anal.* **1**, 2–11 (1979).

[S11] Zangwill, A. *Physics at Surfaces* (Cambridge University Press, 1988).

[S12] Kitchin, J. R., Reuter, K. & Scheffler, M. Alloy surface segregation in reactive environments: First-principles atomistic thermodynamics study of Ag_3Pd(111) in oxygen atmospheres. *Phys. Rev. B* **77**, 075437 (2008).

[S13] Reuter, K. & Scheffler, M. Composition, structure, and stability of RuO_2(110) as a function of oxygen pressure. *Phys. Rev. B* **65**, 035406 (2001).

[S14] Tsuda, Y., Yoshigoe, A., Teraoka, Y. & Okada, M. Surface temperature dependence of oxidation of Cu_3Au(111) by an energetic oxygen molecule. *Mater. Res. Express* **3**, 035014 (2016).

[S15] Okada, M. *et al.* Experimental and theoretical studies on oxidation of Cu-Au alloy surfaces: effect of bulk Au concentration. *Sci. Rep.* **6**, 31101 (2016).

[S16] Ghijsen, J. *et al.* Electronic structure of Cu_2O and CuO. *Phys. Rev. B* **38**, 11322–11330 (1988).

[S17] Okada, M. *et al.* X-ray photoemission study of the temperature-dependent CuO formation on Cu(410) using an energetic O_2 molecular beam. *Phys. Rev. B* **75**, 233413 (2007).

[S18] Derry, G. N. & Ji-Zhong, Z. Work function of Pt(111). *Phys. Rev. B* **39**, 1940–1941 (1989).

[S19] Liao, L. *et al.* Multifunctional CuO nanowire devices: p-type field effect transistors and CO gas sensors. *Nanotechnology* **20**, 085203 (2009).

[S20] Eom, K., Lee, D., Kim, S. & Seo, H. Modified band alignment effect in ZnO/Cu_2O heterojunction solar cells via Cs_2O buffer insertion. *J. Phys. D: Appl. Phys.* **51**, 055101 (2018).

[S21] Siddiqui, H. *et al.* A review: synthesis, characterization and cell performance of Cu_2O based material for solar cells. *Orient. J. Chem.* **28**, 1533–1545 (2012).

[S22] Dhalea, B. B., Mujawarb, S. H., Bhattara, S. L. & Patilc, P. S. Chemical properties of n-ZnO/p-CuO heterojunctions for photovoltaic applications. *Der Chemica Sinica* **5**, 59–64 (2014).

[S23] Fain Jr, S. C. & McDavid, J. M. Work-function variation with alloy composition: Ag-Au. *Phys. Rev. B* **9**, 5099–5107 (1974).

[S24] Fain Jr, S. C. & McDavid, J. M. Work-function variation with alloy composition: Cu-Au. *Phys. Rev. B* **13**, 1853–1854 (1976).

[S25] Ishii, R., Matsumura, K., Sakai, A. & Sakata, T. Work function of binary alloys. *Appl. Surf. Sci.* **169**, 658–661 (2001).

[S26] Pašti, I. & Mentus, S. Electronic properties of the Pt_xMe_{1-x}/Pt(111)(Me= Au, Bi, In, Pb, Pd, Sn and Cu) surface alloys: DFT study. *Mater. Chem. Phys.* **116**, 94–101 (2009).
